# 10-Hydroxy Decanoic Acid-Based Vesicles as a Novel Topical Delivery System: Would It Be a Better Platform Than Conventional Oleic Acid Ufasomes for Skin Cancer Treatment?

**DOI:** 10.3390/pharmaceutics15051461

**Published:** 2023-05-11

**Authors:** Bassant Atef, Rania A. H. Ishak, Sabry S. Badawy, Rihab Osman

**Affiliations:** 1 Department of Pharmaceutics and Industrial Pharmacy, Faculty of Pharmacy, Ain Shams University, Abbassia, Cairo 11566, Egypt; 2 Department of Pharmaceutical Technology, Faculty of Pharmacy, Misr International University, Cairo 12585, Egypt

**Keywords:** fatty acid vesicles, magnolol, ufasomes, topical delivery, 10-hydroxy decanoic acid, oleic acid, skin cancer

## Abstract

10-hydroxy decanoic acid (HDA), a naturally derived fatty acid, was used for the preparation of novel fatty acid vesicles for comparison with oleic acid (OA) ufasomes. The vesicles were loaded with magnolol (Mag), a potential natural drug for skin cancer. Different formulations were prepared using the thin film hydration method and were statistically evaluated according to a Box–Behnken design in terms of particle size (PS), polydispersity index (PDI), zeta potential (ZP), and entrapment efficiency (EE). The ex vivo skin permeation and deposition were assessed for Mag skin delivery. In vivo, an assessment of the optimized formulae using 7,12-dimethylbenz[a]anthracene (DMBA)-induced skin cancer in mice was also conducted. The PS and ZP of the optimized OA vesicles were 358.9 ± 3.2 nm and −82.50 ± 7.13 mV compared to 191.9 ± 6.28 nm and −59.60 ± 3.07 mV for HDA vesicles, respectively. The EE was high (>78%) for both types of vesicles. Ex vivo permeation studies revealed enhanced Mag permeation from all optimized formulations compared to a drug suspension. Skin deposition demonstrated that HDA-based vesicles provided the highest drug retention. In vivo, studies confirmed the superiority of HDA-based formulations in attenuating DMBA-induced skin cancer during treatment and prophylactic studies.

## 1. Introduction

Skin cancer, specifically non-melanoma skin cancer, is the most widely diagnosed type of cancer in Caucasian populations. Basal cell carcinoma (BCC) represents 70%, while squamous cell carcinoma (SCC) constitutes 25% of the non-melanoma skin cancer [1]. The mechanism of skin carcinogenesis is still under investigation. Molecular and genetic alterations were reported as the leading cause of non-melanoma skin cancer. Other risk factors are also involved, such as; age, ionizing radiation, carcinogenic chemicals, human papillomavirus, UV radiation and immunosuppression [2]. Due to the rising incidence of non-melanoma skin cancer, it is crucial to focus on improving its management, prevention, early screening, and diagnosis. Topical treatment with chemotherapeutics was suggested for superficial skin cancer due to its benefits in increasing drug localization into the site of action and reduced side effects compared to systemic treatment. [3,4,5]. Developing new options with natural products with no reported side effects could be of great benefit in controlling non-melanoma skin cancer [6].

**Figure 1:**
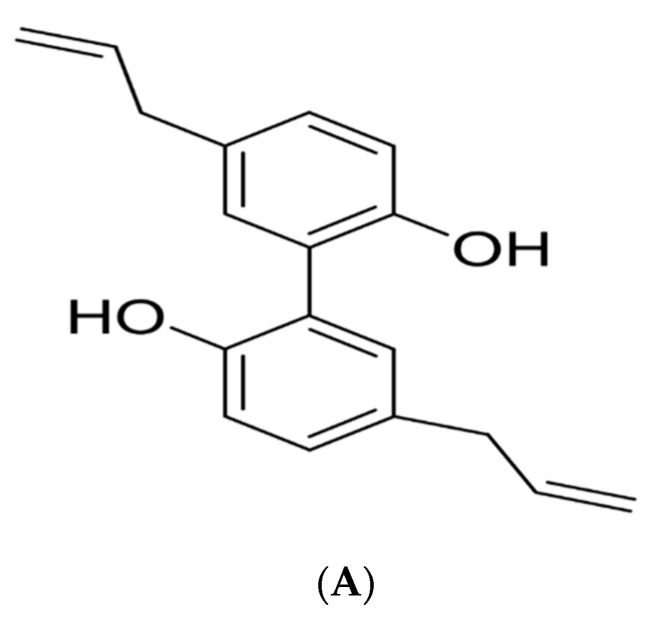

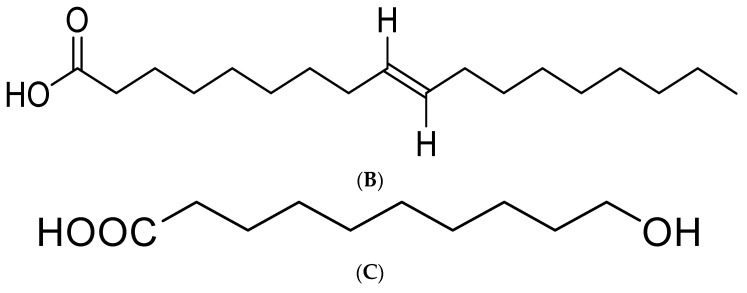
Chemical structure of (**A**) magnolol (Mag), (**B**) Oleic acid (OA), (**C**) 10-hydroxy decanoic acid (HDA).

Magnolol (Mag) is a lignan isolated from the stem bark and root of *Magnolia officinalis*; its chemical structure is illustrated in Figure 1A. Mag manifests various pharmacological effects, including anti-oxidative, muscle relaxant, anti-atherosclerosis, anti-microbial, anti-inflammatory and anti-cancer effect [7,8]. The anti-tumor effects of Mag against different types of cancers, such as lung cancer, breast cancer, hepatocellular carcinoma, colon cancer, esophagus cancer and skin cancer, had been previously reported [6,8,9,10,11,12,13]. Mag’s ability to enhance apoptosis, and induce G2/M phase cell cycle arrest, added to its potential effects on several pathways, are the possible mechanisms responsible for its activity in the skin cancer [13,14].

The poor Mag aqueous solubility (log P = 3.94) [15], limited oral absorption caused by significant hepatic metabolism and high plasma protein binding pose challenges for Mag oral delivery [16]. Moreover, Mag’s poor stability and incompatibility with strong oxidizing agents is one of the drawbacks that can evolve during the formulation [17]. Therefore, topical delivery of Mag to its site of action using tailored nanocarriers was suggested to ensure its efficacy in the treatment of skin cancer.

Unsaturated fatty acid vesicles or ‘ufasomes’ are colloidal dispersions that were first prepared by Gebicki and Hicks in 1973 using oleic acid Figure 1B. Their negatively charged soaps are organized in closed lipid bilayers with the hydrocarbon tails pointing towards the interior of the membrane, and the carboxyl groups (head) are in contact with the aqueous core. Therefore, the stability of the vesicles depends on the ratio between the fatty acid ionized form and its neutral form [18,19]. Ufasomes provide a prolonged drug release and hence, reduce side effects, including skin irritation, skin scaling, swelling and inflammation caused by repetitive application of topical medications.

10-Hydroxydecanoic acid (HDA) is the queen bee fatty acid recognized as the major component of Royal jelly (RJ). RJ is released by the nurse bees’ cephalic glands. It provides the queen bee with all her nutritional needs throughout her lifetime, and it has been extensively used in cosmetics, medical products and dietary supplements. The chemical formula of HDA is C_10_H_20_O_3,_ and its chemical structure is depicted in Figure 1C. HDA, with a log *P* (o/w) value of 1.847, is soluble in organic solvents, including methanol and chloroform. Based on many studies, HDA has been found to be responsible for most of the pharmacological effects of RJ, including; anti-inflammatory, wound healing, anti-oxidant, anti-tumor, anti-bacterial, anti-allergic, anti-fatigue, anti-hypercholesterolemia, anti-aging, general tonic and insulin-like properties [20,21,22,23]. Due to its promising beneficial effects on the skin, it was used, in this work, as a potential novel multi-functional carrier, instead of OA in the preparation of novel fatty acid vesicles.

The purpose of the current study was to deliver Mag to skin cancerous cells through tailored nanocarriers, such as ufasomes and innovative HDA-based vesicles. Fatty acid vesicles were adopted for Mag loading to enhance its stability, permeation and skin deposition for the management of DMBA-induced skin cancer.

## 2. Materials and Methods

### 2.1. Materials

10-Hydroxy decanoic acid (HDA) (purity > 95%) was purchased from Wuhan Yuancheng Gongchuang Technology Co., Ltd. (Wuhan, China). Magnolol (Mag) (purity > 99%) was purchased from Xian Lyphar Biotech Co., Ltd. (Xi’an, China). Trizma^®^ base or Tris(hydroxymethyl)aminomethane (Tris) and phosphoric acid were purchased from Sigma-Aldrich Chemical Co. (St. Louis, MO, USA). Acetonitrile and methanol (HPLC grade) were provided by Merck Chemicals, Germany. Chloroform and methanol were supplied from El-Nasr Chemical Co., Cairo, Egypt. Disodium hydrogen phosphate and dihydrogen sodium phosphate were purchased from Piochem Co., Egypt. H&E stain was purchased from Merck, Germany. Oleic acid (OA), Span^®^ 80 and Tween^®^ 80 were purchased from Loba Chemie Pvt, Ltd., India. Nanosep^®^ centrifugal filters: Centrifuge tube fitted with an ultra-filter with MWCO 100 KDa was purchased from Pall Life Science, Port Washington, NY, USA. The dialysis membrane of 14 KDa was bought from Spectrum Laboratories Inc., San Francisco, CA, USA. Paraffin oil, formalin, xylene, and ethanol were supplied from El-Nasr Chemical Co., Cairo, Egypt. 7,12-dimethylbenz(a)anthracene (DMBA) was purchased from Sigma-Aldrich Chemical Co. (St. Louis, MO, USA). Malondialdehyde (MDA) assay kit was bought from Eagle Biosciences, Boston. Glutathione (GSH) assay kit was purchased from Cayman Chemicals, USA. Anti-Ki-67 antibody was purchased from Abcam, UK.

### 2.2. Methods

#### 2.2.1. Preparation of Plain Fatty Acid Vesicles

The preparation of ufasomes using oleic acid (OA) and HDA vesicles using 10-hydroxy-decanoic acid (HDA) was performed using the thin film hydration approach, with slight modifications [19,24]. For the preparation of the plain vesicles, the calculated amount of fatty acid and surfactant was added to a mixture of methanol and chloroform prepared at a 1:1 (*v*/*v*) ratio and was completely dissolved by sonication for 1 min in a dry round bottom flask. The organic solvents were then allowed to evaporate using a rotary evaporator adjusted at a rotational speed of 120 rpm for 40 min and temperatures of 65 and 80 °C for OA and HDA, respectively, till a thin film was developed. The film was subsequently hydrated using 10 mL Tris buffer (pH 8 ± 0.1) for 20 min. Finally, the prepared formulae were subjected to sonication in a bath sonicator for 30 min to ensure uniform particle size (PS) and minimize aggregation. The prepared dispersions were then stored under refrigeration at 5 ± 3 °C for subsequent characterization.

#### 2.2.2. Preparation of Mag-Loaded Nanovesicles

For the preparation of Mag-loaded vesicles, a stock solution of Mag in methanol at a concentration of 5 mg/mL was first prepared and added to the fatty acid-surfactant mixture in a dry round bottom flask. The procedure previously described in Section 2.2 was then followed. The prepared formulations were kept at 5 ± 3 °C for subsequent characterization [19,25,26].

#### 2.2.3. Optimization of Mag-Loaded Vesicular Formulations

A Box–Behnken statistical design was implemented for optimizing the formulation parameters of the prepared OA and HDA vesicles; three numerical factors at three levels were assessed for each fatty acid used. The investigated independent variables were: the concentrations of fatty acid (A), surfactant (B), and Mag (C) for each fatty acid type (D). Table 1 displays the low, medium, and high levels of each factor. Thirteen formulation runs, with three central points (of the same composition to check error), were prepared accordingly for each fatty acid type. The particle size (PS, Y_1_), polydispersity index (PDI, Y_2_), the surface charge expressed as zeta potential (ZP, Y_3_), and the drug entrapment efficiency (EE, Y_4_) were considered as the responses.

#### 2.2.4. Characterization of the Prepared Fatty Acid Vesicles

All the prepared Mag-loaded formulations were characterized as described below.

##### PS and PDI Measurements

The Malvern Zetasizer apparatus was used to measure PS using the dynamic light scattering (DLS) technique. All fresh formulations were diluted with distilled water to reach a count rate of 200–400 kilo count per second (KCPS), and the measurements were carried out at 25 °C. The results of the Z-average PS (in nm) and PDI were determined in triplicate for each formulation, and the average data was calculated [27,28].

##### Zeta Potential (ZP) Determination

The surface charge expressed as ZP (in mV) of the prepared fatty acid nanoparticles was measured based on their electrophoretic mobility using Zeta cells in the Malvern Zetasizer instrument after suitable dilution with distilled water before analysis. The mean of triplicate results was calculated for each formulation [27].

##### Determination of Mag Encapsulation Efficiency (EE)

The indirect method was used for the determination of the Mag EE % [25]. Briefly, an accurate volume of 25 µL of each prepared formulation was placed in the upper compartment of the Nanosep^®^ centrifugal filters, then diluted with 475 µL of filtered distilled water. Centrifugation was performed for 60 min using a cooling centrifuge adjusted at 7000 rpm and 4 °C. After complete separation; the supernatants were analyzed spectrophotometrically at the predetermined λ_max_. To compute the EE, the following equation was applied:

(1) $$\mathrm{EE}=\frac{C\left(T\right)-C\left(S\right)\;}{C\left(T\right)}\times 100\;$$

where C(T) and C(S) are the initial and measured Mag concentrations in the supernatant, respectively. The mean value for three replicate determinations was recorded.

##### Morphological Examination by Transmission Electron Microscope (TEM)

Freshly prepared optimized Mag-loaded formulations were imaged by TEM. A drop of the selected ufasomal dispersion was placed on a carbon-coated copper grid, negatively stained with 1% phosphotungstic acid, and then allowed to dry at room temperature for 10 min prior to examination. The sample was imaged using a JEOL 2100 TEM operating at 200 kV, and real-time images were recorded with a 500 ms exposure time. The fresh sample was continuously delivered to the tip of the holder. All dispersions were observed at different magnification powers [27,28].

##### Differential Scanning Calorimetry (DSC) Analysis

The thermal behaviors of Mag, HDA, plain ufasomes, plain HDA vesicles and optimized Mag-loaded formulations were examined. A fixed amount of each powder (1 mg) was placed in an aluminum pan followed by folding, noting that all formulations were air dried in an aluminum pan overnight till powder formation. Over a temperature range of 20 to 300 °C, the thermogram was plotted at a nitrogen gas flow rate of 20 mL/min and a heating rate of 10 °C/min [24].

### 2.3. In Vitro Drug Release Study

To compare the release profile of optimized Mag-loaded vesicles to the free drug suspension, the previously described non-equilibrium dialysis method was used with a minor modification [29]. Briefly, an own-designed cell composed of two compartments was fabricated. The donor compartment was filled with the test formulation, while the receptor compartment contained 25 mL of dissolution medium (PBS, pH 7.4 containing 2% *w*/*v* Tween^®^ 80). The two compartments were separated by a dialysis membrane (molecular weight cut-off diameter 14 KDa), and the cell temperature was set at 37 °C. At fixed time intervals, 0.25, 0.5, 1, 2, 4, 6, 8, 24 and 48 h, a sample of 1 mL was taken out of the receptor compartment and was replaced with an equivalent volume of fresh dissolution medium. The drug concentration was determined by spectrophotometry at the predetermined λ_max_ using the constructed calibration curve. The concentration was converted to a percentage cumulative drug release and was then drawn against time, constructing the release profile [30].

### 2.4. Ex Vivo Skin Permeation and Deposition Studies

#### 2.4.1. Preparation of Rat Skin

The experimental protocol was approved by the Ethics Committee, Faculty of Pharmacy, Ain Shams University (REC approval # 140). Healthy male rats weighing 200 ± 20 g were chosen for permeation experiments; their hairs were shaved without damaging the abdominal skin area. The rats were euthanized by cervical dislocation. A surgical scalpel was used to separate the abdominal skin, and then the underlying subcutaneous tissue and fats were stripped away cautiously. The skin was cleaned with normal saline and packed in aluminum foil at −20 °C. Before use, each skin specimen was carefully checked for integrity.

#### 2.4.2. Skin Permeation Studies

The skin permeation of the optimized formulations was studied through the excised rat skin using our own-fabricated cells. The available diffusion area was 5.7 cm^2^. The full-thickness rat skin was fixed between the donor and receptor compartments so that the side of the stratum corneum of the skin was facing the donor compartment. The receptor chamber was filled with 25 mL of PBS (pH 7.4) containing Tween^®^ 80 (2%, *w*/*w*) to achieve sink condition. The cell was placed in a shaking water bath set at 45 rpm, and its temperature was maintained at 37 °C [31] till the end of the experiment. A specified weight of Mag-loaded vesicular formulation, equivalent to the calculated specified amount of Mag, was applied onto the skin in the donor compartment side. At specific time intervals of 0.25, 0.5, 1, 2, 4, 6, 8, 12, and 24 h, a 1-mL sample was withdrawn from the receiving medium and instantly replaced with an equal volume of fresh dissolution solution. The amount of drug in the samples was analyzed using the HPLC method described earlier [29]. The cumulative amount of Mag permeated per unit area was calculated using the following equation [32]:

(2) $$Q_{n}=\frac{V_{o}\;C_{n}+\sum_{i=1}^{n-1}C_{i}\;\mathrm{Vs}}{A}$$

where Q_n_ (μg/cm^2^) is the cumulative amount of the drug permeated per unit area at different sampling times, C_n_ is the drug concentration of the solution in the receptor compartment at each sampling time, C_i_ is the drug concentration of the *i*th sample, Vs is the sample volume (Vs = 1 mL), V_o_ is the volume of solution in the receptor compartment *(*V_o_ = 25 mL)*,* respectively, and A is the effective diffusion area (A = 5.7 cm^2^).

A plot of the cumulative amount permeated vs. time was created, and the steady state flux (Jss) was then computed from the slope of the linear part of the graph and enhancement ratio (ER) was calculated from the following equations [33,34]:

(3) $$\mathrm{ER}=\frac{\mathrm{Flux}\;\mathrm{of}\;\mathrm{ufasomal}\;\mathrm{formulation}\;}{\mathrm{Flux}\;\mathrm{of}\;\mathrm{free}\;\mathrm{drug}\;\mathrm{suspenion}\;}$$

#### 2.4.3. Skin Deposition Studies

The skin was separated from the cell after 24 h of the permeation study and thoroughly washed with filtered distilled water. The excised skin was placed in a falcon tube in PBS pH 7.4 containing 2% Tween^®^ 80 and methanol (HPLC grade) at a ratio of 1:1 *v*/*v*. Then the tube content was homogenized and centrifuged at 3000 rpm, 4 °C for 15 min. The samples were filtered with 0.22 µm disposable filters and then analyzed for drug content by HPLC. The percent of drug retained in the skin was calculated [24].

### 2.5. Physical Stability Study of Mag-Loaded Nanovesicles

To investigate how storage affects the preparations’ physical stability, the optimized OA and HDA vesicular dispersions were stored under refrigeration at 5 ± 3 °C for 4 months, and the various formulation parameters PS, PDI and EE were measured initially and at 1-month intervals for 4 months [34].

### 2.6. In Vivo Anti-Cancer Evaluation

Chemically induced skin carcinogenesis in animal models is the main model for cutaneous carcinogenesis in mice or rats and is important for understanding the multistage process of epithelial cancers and for evaluating the anti-cancer effects of various drugs [4,35]. DMBA or 7,12-dimethylbenz[a]-anthracene is one of the most common carcinogens used for cutaneous cancer induction [36]. DMBA-induced skin cancer mice model was, thus, employed to evaluate the efficacy of the natural anti-cancer agent (Mag) and to assess the potential of the optimized Mag-loaded formulations in the prevention and treatment of cutaneous cancer.

#### 2.6.1. Animal Model

Male albino mice (20–25 g) were used to establish the skin tumor animal model. The experimental protocol was approved by the Ethics Committee, Faculty of Pharmacy, Ain Shams University (REC approval # 140). The animals were kept in an animal housing facility at a temperature of 25 °C with a regular 12-h light/dark cycle in plastic cages. The mice received unlimited access to food and water. The total number of mice at the beginning of the experiment was 90, divided into 6 groups (n = 15 per group). In all mice, a 3 × 3 cm^2^ dorsal skin area was shaved 48 h before the beginning of induction. The six animal groups were classified as follows; Groups I and II were the respective negative and positive controls. Groups III, IV and V received treatment F-O1, F-O2 and F-O3, respectively. Group VI received prophylactic treatment with F-O2 every other day, 7 days *prior* to beginning the induction and continued during the induction and treatment period. The skin tumor was induced in mice of the positive control group without any treatment, while the negative group of mice was not subjected to any induction or treatment protocol.

#### 2.6.2. Tumor Induction Using DMBA

First, an accurately weighed amount (125 mg) of DMBA was suspended in 25 mL paraffin oil. This suspension was applied topically onto the skin by painting directly an amount equivalent to 0.5 mg/0.1 mL paraffin/ mouse on the dorsal side of mice (for Groups II to VI) every other day for 2 months. The shaved area was inspected weekly for the appearance of any tumors.

#### 2.6.3. Treatment with the Selected Mag-Loaded Formulations

One month after starting tumor induction, each of the three treatment groups received different formulations: F-O1, F-O2 and F-O3 to Group III, Group IV and Group V, respectively, all at a specific drug dose of 15 mg/kg/day [16,37] by direct topical application of the formulation every other day for 30 days. Group VI represents the prophylactic group to which the selected formula (F-O2) was applied 7 days before induction, during the 30-day induction, and 30 days after induction.

During treatment, the tumor size was inspected weekly and measured using a caliper till the end of the study. Then the mice were euthanized by cervical dislocation, and tumor samples were excised and stored in a 10% formalin solution for subsequent analysis.

#### 2.6.4. Assessment of Mice Body Weight

Body weight monitoring was used as an indicator of the overall health status of mice. Weekly assessment of body weight using electrical balance was performed, and the average data per group was recorded [38].

#### 2.6.5. Measurements of Tumor Size and Number of Papilloma

Tumor incidence was recorded, and only papillomas with more than 1 mm diameter were recorded. A caliper was used to measure the tumor’s size, and the number of papillomas was recorded [38].

#### 2.6.6. Determination of Malondialdehyde (MDA) and Glutathione (GSH) Levels in Skin Tumor Samples

MDA is a product of lipid peroxidation detected in high levels after exposure to increased oxidative stress [39]. GSH is one of the common antioxidants present in cells [40]. Detection and quantification of both markers provide an indication of the anti-cancer activity of the applied treatment. After 30 days of continuous treatment with the selected formulations, the excised tumor samples were cleaned with water and homogenized, centrifuged and filtered for MDA and GSH analysis by UV-visible spectrophotometer at λ_max_ = 586 nm and 412 nm, respectively.

#### 2.6.7. Immunohistochemistry (Ki-67 Expression) Study

Ki-67 is a proliferation marker that is frequently employed in many cancer types [41,42,43]. Skin samples were removed, promptly fixed in 10% neutral formalin solution, properly rinsed under running water, dehydrated using successive rising ethanol dilutions, and then placed in xylene for complete alcohol clearance. The skin specimens were fixed in paraffin wax, dissected into segments that were 4-μm thick, and mounted on glass slides for immuno-peroxidase staining. This staining technique used anti-Ki-67 antibodies to detect Ki-67 in the skin tissue. The selected specimens were examined by light microscopy [44].

#### 2.6.8. Histopathological Examinations

For histological examinations, H&E stain was employed for light microscopy because of its ability to distinguish a vast range of normal and abnormal tissue components. The excised skin specimens were prepared as described above. The skin specimens were fixed on glass slides for H&E staining after being immersed in paraffin wax and sectioned into 4-m-thick blocks by a microtome. Histopathological examinations were performed under a light microscope [27,44,45].

### 2.7. Statistical Analysis

All formulations were prepared in triplicates. Results are expressed as mean ± SD (standard deviation). All in vivo experiments and measurements were carried out on all six groups (n = 15) per group, and biomedical tests were carried out on six mice from each group (n = 6). Results are expressed as mean ± SD (standard deviation). One-way ANOVA was used to test the differences between treatments, and Tukey’s test was performed for multiple comparisons. A *p*-value ≤ 0.05 was taken as significant.

All experimental data obtained according to the established Box–Behnken design were analyzed and optimized statistically using Design-Expert^®^ software (Version 7, Stat-Ease Inc., Minneapolis, MN, USA).

To check the validity of equation models, a few vesicular formulations were randomly chosen as the confirmatory checkpoints. Their experimental results were then compared to the predicted ones. The prediction error was accordingly computed from the following equation:

(4) $$\mathrm{Prediction}\;\mathrm{error}=\frac{\mathrm{Predicted}\;-\mathrm{Experimental}}{\mathrm{Experimental}}\times 100$$

## 3. Results and Discussion

### 3.1. Preparation of Plain Fatty Acid Vesicles

Ufasomes and HDA vesicles were prepared by the thin film hydration technique, one of the most popular techniques reported for the preparation of multilamellar vesicles, because of its simplicity and suitability for all lipid types [46].

To ensure the proper assembly of fatty acid vesicles, the rotational speed of the rotary evaporator is one of the critical factors for vesicles formation, hence the speed of evaporation and hydration was adjusted at 120 rpm as reported by other researchers as lower and higher values are often responsible for aggregates formation [34].

Also, the organic solvent should be evaporated at a temperature higher than the chain melting temperature (Tm) of each fatty acid so that uniform thin films free from aggregates and clumps are formed [18]. Therefore, the temperature was adjusted at 65 °C for OA vesicles as previously reported [24,27], but in the case of HDA, it was maintained at 80 °C, a temperature higher than the reported fatty acid Tm (75–77 °C) [47,48].

The hydration of the formed film at a suitable pH is also essential for vesicle formation. The pH used should allow for the ionization of the fatty acid carboxylic groups to ensure the formation and stability of fatty acid vesicles. Hence, the pH was adjusted at 8, a value equal to the pKa of OA, to ensure the ionization of half of the fatty acid carboxylic groups [25,27,28]. However, HDA-based vesicles were successfully prepared at the same pH producing stable vesicles although the HDA pKa value is equal to 5.5 [49]. This was in agreement with Novales et al., who reported the preparation of self-assembled vesicles composed of hydroxyl saturated fatty acids as 12-hydroxystearic acid and omega-hydroxy palmitic acid at pH (8.5–9) much higher than the fatty acids pKa value (=4.95). This was attributed to the formation of hydrogen bonding between the -OH groups of these fatty acid molecules inducing steric hindrance within the alkyl hydrophobic layer and hence increasing the free energy of their crystal state [50].

### 3.2. Optimization of Mag-Loaded Vesicles According to Box–Behnken Design

Mag-loaded fatty acid vesicles were prepared using the thin film hydration technique by applying the statistical Box–Behnken design. The formulation variables and their levels were set based on the results of the preliminary studies. The effect of different formulation variables, namely; fatty acid concentration (A), surfactant concentration (B), and drug concentration (C), each at three levels for each fatty acid type (D), were investigated by generating 30 formulae with three central points of the same composition to check error. The measured responses were PS (Y1), PDI (Y2), ZP (Y3) and EE (Y4) of all prepared formulations of varied compositions. Based on the results of Table 2, the OA and HDA-based vesicles were successfully prepared where their PS ranged between 156.40 ± 1.56 and 836.60 ± 397.90 nm with size distribution lying from 0.168 ± 0.014 to 0.790 ± 0.231. The surface charges of all prepared vesicles revealed high negative magnitudes of ZP values varying from −44.1 ± 7 to −90.1 ± 3 mV. A high drug entrapment was observed in all loaded formulae as the calculated EE data surpassed 63% to almost complete drug loading (~97%).

#### 3.2.1. Model Generation

ANOVA test results in Table 3 revealed significant models for PS, ZP and EE responses (*p* < 0.05) with F-values of 165.82, 23.65 and 143.77, respectively. The F-values of “Lack of Fit” were 3.03, 1.75 and 1.07 for the respective responses implying a non-significance relative to the pure error. Noting that the PDI model was statistically found non-significant (*p* > 0.05). The respective R^2^ values recorded in Table 4 confirmed the agreement of the predicted values calculated from the three generated predictive models with their actual results.

#### 3.2.2. PS Response

ANOVA test results confirmed that all studied independent variables revealed significant effects (*p* < 0.05), yet with variable magnitudes on the PS of the prepared vesicular formulations. The concentrations of both fatty acid (A) and drug (C) demonstrated the highest impacts on vesicle sizes, while the surfactant concentration and the fatty acid type were the least as delineated from their corresponding F-values.

##### Effect of Fatty Acid Concentration on PS

**Figure 2:**
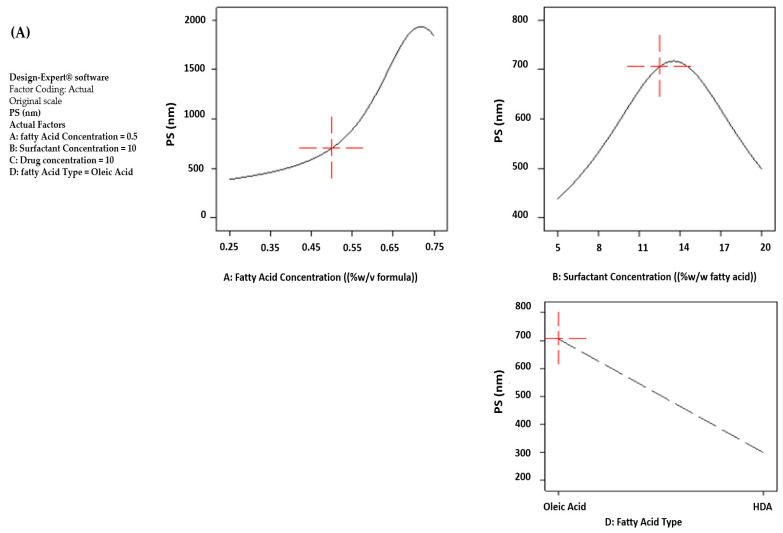

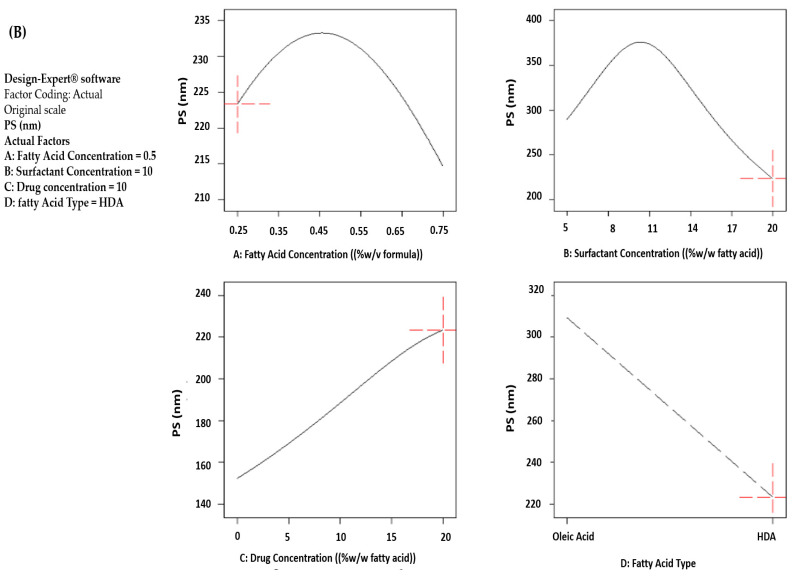

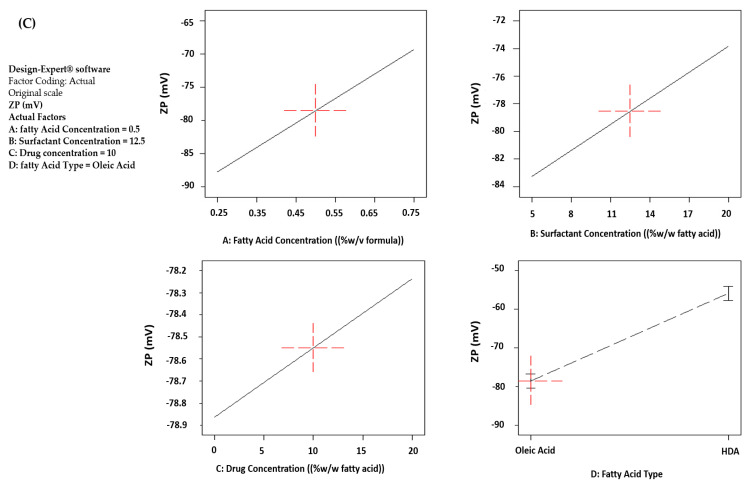

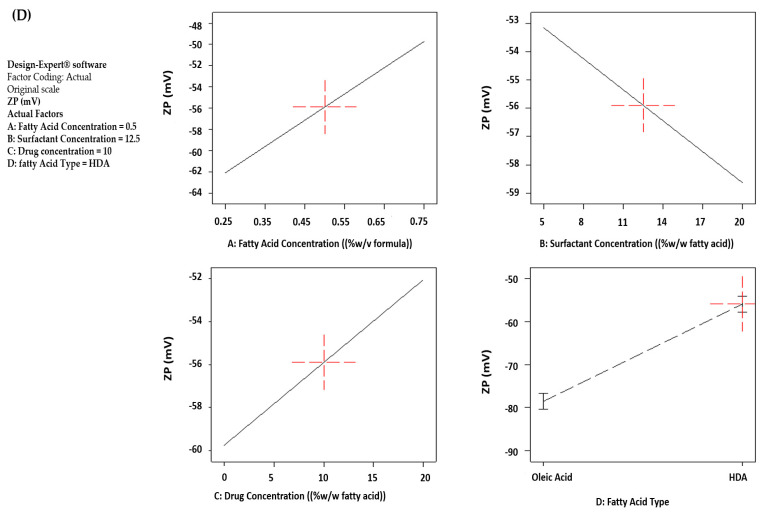

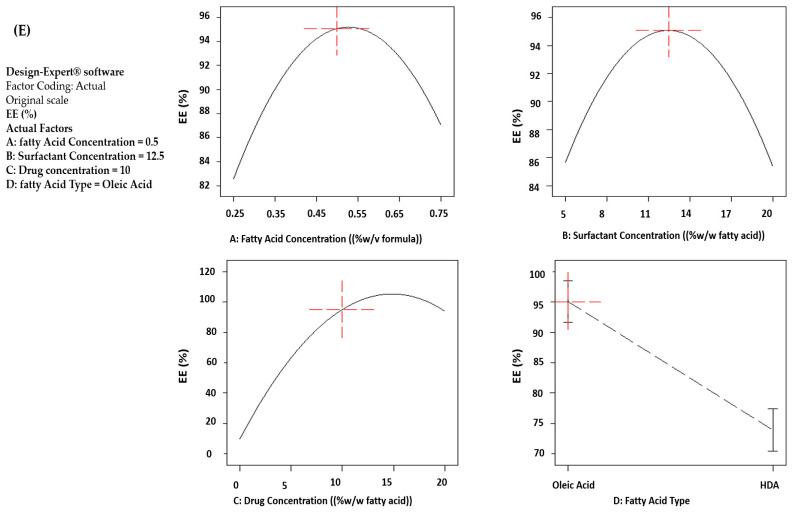

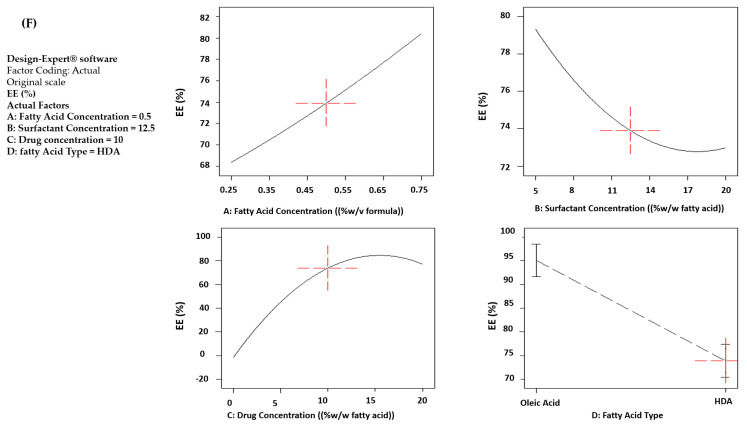
Main effect plots of significant independent variables on (**A**) PS of OA-based vesicles, (**B**) PS of HDA-based vesicles, (**C**) ZP of OA-based vesicles, (**D**) ZP of HDA-based vesicles, (**E**) EE of OA based vesicles, and (**F**) EE of HDA-based vesicles prepared according to Box–Behnken design.

The main effect plots, illustrated in Figure 2A,B, revealed that the average PS of the formed vesicles increased exponentially by raising OA concentration from 0.25 to 0.75% (*w*/*v*). It is to be noted that high concentrations of OA (0.75%) led to particles with high standard deviation values. These results are well correlated with [51,52] because higher OA concentration resulted in the formation of larger bilayer vesicles aiming at reducing the exposure of hydrocarbon chains to water [31]. Guo and co-workers also reported that high concentrations of lipids resulted in increased vesicle flexibility with consequent larger PS due to their ability to include more molecules [53]. On the other hand, HDA-based vesicles revealed a parabolic size pattern when the rising HDA concentration was from 0.25 to 0.75%, with a size peak at 0.5%. This might be attributed to the formation of smaller size micelles instead of vesicles at higher concentrations of HDA (0.75%). A similar observation had been reported in a previous work where dexamethasone-loaded OA vesicles exhibited the same pattern [36].

##### Effect of Mag Concentration on PS

Mag concentration significantly affected the PS of HDA-based vesicles (*p* < 0.05), where a positive correlation was observed between PS and drug concentration, as demonstrated in Figure 2B. The inclusion of Mag was associated with an expansion in vesicular size from 199.50 ± 1.77 to 235.80 ± 11.74 nm and from 477.80 ± 10.04 to 752.80 ± 5.37 nm as revealed from formulations F18 and F15, F25 and F11 when increasing drug content from 0 to 20% at the same fatty acid and surfactant concentrations. These results could be related to the fact that at higher drug concentrations, the vesicular sizes are expected to increase where the vesicles could expand to contain more drugs. Similar results were reported with [51,54]. On the contrary, the size of OA-based ufasomes remained unchanged irrespective of Mag concentration. The corresponding OA formulations, F5 and F8, F23 and F24, confirmed this finding as their respective sizes; 547.85 ± 155.35 and 324.20 ± 36.20 nm, 437.60 ± 11.40 and 477.80 ± 10.04 nm were considered non-significantly different at 0 and 20% drug concentration (*p* > 0.05). This could be due to the high capacity of OA vesicles to encapsulate this range of drug concentration without affecting the size of the vesicles. Although comparing some OA-based formulae might reveal the opposite, yet, by taking into consideration the other formulae and the high SD scored with some formulae, the statistical analysis of the implemented design, as presented in ANOVA data (Table 3) and the main effect plots in Figure 2, delineate the non-significance of the observed difference.

##### Effect of Span^®^80 Concentration on PS

Surprisingly, the effect of surfactant concentration on PS of the prepared vesicles showed a parabolic contour whatever the type of fatty acid used, as illustrated in Figure 2A,B. That’s to say, the smallest vesicle sizes were attained at both level extremities of Span^®^80 concentration (5 and 20%), while at the mid-level of surfactant concentration (12.5%), larger vesicle sizes were unexpectedly produced. Increasing surfactant concentration from 5 to 20% demonstrated a non-significant change in PS of OA-based ufasomes (*p* > 0.05). This was confirmed by formulae F14 and F7, F5 and F24, F17 and F26 with respective sizes 344.37 ± 43.88 and 362.30 ± 21.92 nm, 547.85 ± 155.35 and 477.80 ± 10.04 nm, 495.60 ± 25.17 and 432.53 ± 125.53 nm when augmenting Span^®^ concentration from 5 to 20% at equivalent OA and drug concentrations. Similarly, the HDA-based formulae F30 and F12, F25 and F18 showed non-significant small sizes of 185.10 ± 7.88 and 185.80 ± 17.96 nm, 167.90 ± 13.01 and 199.50 ± 1.77 nm at 5 and 20% surfactant concentrations, respectively (*p* > 0.05).

##### Effect of Fatty Acid Type on PS

As demonstrated in Figure 2A,B, shifting the fatty acid type from OA to HDA significantly reduced the PS of the prepared vesicles (*p* < 0.05). This was confirmed by the lower PS range of HDA-based vesicles compared to those prepared with OA; 156.40 ± 1.56 to 481.05 ± 47.87 nm versus 291.55 ± 49.55 to 836.60 ± 397.9 nm, respectively. The smaller vesicular sizes obtained with HDA could be ascribed to the shorter length of its hydrocarbon chain (C9) compared to the long linear carbon chain of OA (C17) [55,56].

##### Two-Way Interactions

**Figure 3:**
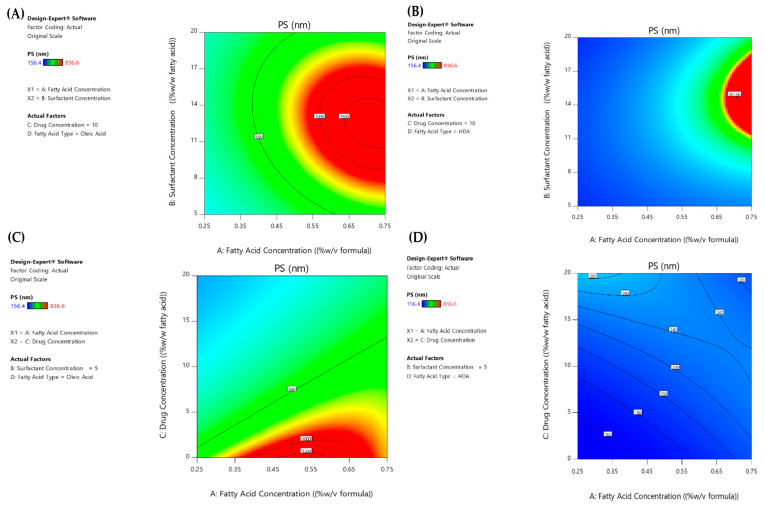
Two-way interaction contour plots of PS response of (**A**,**C**) OA and (**B**,**D**) HDA-based vesicles.

Based on ANOVA test results presented in Table 3, it is evident the significant effects of several two-way interactions (2-FI), AB, AC, AD, BC, BD and CD, on PS of the formed fatty acid vesicles. The 2-FI contour and normal plots of these interactions are collected and illustrated in Figure 3.

By referring to Figure 3A,B, it is clear that the lowest PS can be produced at the lowest fatty acid concentration (A: 0.25%) irrespective of the surfactant concentration (B) in the case of OA ufasomes. However, the small-sized had vesicles are only formed on two occasions: (1) at the lowest surfactant concentration (B: 5%), whatever the fatty acid concentration (A), and (2) at the highest surfactant concentration (B: 20%) and the lowest fatty acid concentration (A: 0.25%).

As observed from AC interaction plots shown in Figure 3C,D, the small-sized OA nanostructures can be formed at the highest drug concentration (20%) for all fatty acid concentrations. Contrary, HDA-based vesicles demonstrated convenient smaller sizes (<300 nm) at all fatty acid (A) and drug (C) concentrations studied.

As expected, the plain vesicles prepared with HDA at concentrations (0.25 and 0.5%) showed lower PS compared to loaded ones; this confirmed that the drug retained in lipid bilayers was responsible for the large vesicle size. Surprisingly, drug-loaded HDA vesicles (F28 = 243.20 ± 37.62 nm) scored significantly small sizes compared to those of plain vesicles (F4 = 413.20 ± 55.50 nm) at the highest fatty acid concentration (0.75%), Figure 3D. This could be referred to as the destabilized vesicles with possible drug leakage at the high fatty acid concentration (0.75%), leading to smaller vesicle size [31].

Furthermore, it is evident from Figure 3C,D, that the vesicle sizes depended on both the concentration (A) and type (D) of fatty acids. For instance, no significant change was noted in vesicle sizes prepared with HDA at different lipid concentrations (*p* > 0.05), while decreasing OA concentration from 0.75 to 0.25% revealed a significant PS reduction of the vesicles (*p* < 0.05).

The BC, BD and CD interactions all exhibited varied influential effects on the PS of the produced vesicles. Contrary to what is well known, the increase in surfactant concentration (B) did not induce a size reduction under all circumstances, only at 0 and 20% Mag concentrations (C) in the case of OA- and HDA-based vesicles (D), respectively. It can also be deduced that the smallest sizes of loaded OA ufasomes were attainable irrespective of surfactant concentration; however, for HDA vesicles, only the highest Span^®^ concentration (20%) was capable of forming small-sized medicated vesicles. 

#### 3.2.3. ZP Response

The surface charges of vesicles, expressed as ZP value, are considered a vital parameter for the stability of the formed nano-dispersions. Higher ZP magnitudes, both above +30 mV or below −30 mV, give a prediction of the colloidal stability of the fatty acid vesicles by warranting the repulsion between vesicles and hence preventing flocculation and aggregation [26]. It is worth noting that all experimental runs acquired high magnitudes of ZP values ranging from −44.1 ± 7 to −90.1 ± 3 mV.

From Table 3 of ANOVA test results, we can observe the prominent effect of both fatty acid concentration (A) and type (D) on the surface charges of the formed vesicles (*p* < 0.05), yet the type of fatty acid is the most influential factor acquiring the highest F-value of 168.48. The influence of the concentrations of both surfactant (B) and drug (C) was found non-significant on ZP values of the prepared fatty acid vesicles (*p* > 0.05). The effects of all independent variables on ZP are illustrated in the main effect plots shown in Figure 2C, and D for OA- and HDA-based vesicles, respectively.

##### Effect of Fatty Acid Type on ZP

Changing the fatty acid type from OA to HDA is accompanied by a significant reduction in the negative magnitudes of ZP values (*p* < 0.05), where the respective average results are −80 and −55 mV, as revealed in Figure 2C,D. This could be ascribed to the difference in pKa values of the two fatty acids where the majority of carboxyl groups of HDA, in the presence of Tris buffer, were neutralized, which decreased its electronegativity, compared to OA, where half ionization of its -COOH groups occurred under the same basic conditions [30,49].

##### Effect of Fatty Acid Concentration on ZP

Unexpectedly, increasing the fatty acid concentration from 0.25 to 0.75% resulted in a significant decrease in the average absolute ZP values from −87 to −70 mV and from −62 to −50 mV in the case of OA- and HDA-based vesicles respectively, (*p* < 0.05). The increase in the amount of fatty acid available probably resulted in vesicle coalescence, hiding some of the negative charges present on the particle surfaces.

##### Two-Way Interactions

**Figure 4:**
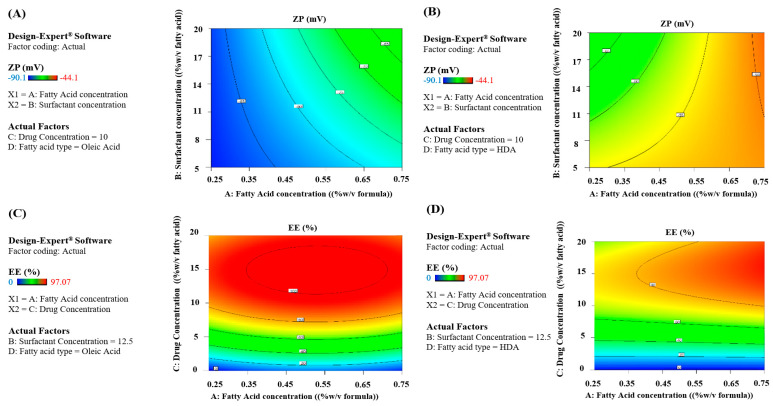
Two-way interaction contour plots of ZP response of (**A**) OA and (**B**) HAD-based vesicles and EE response of (**C**) OA and (**D**) HDA-based vesicles.

By referring to Table 3, only AB and BD interactions exhibited significant effects on the surface charges of the prepared fatty acid vesicles. As depicted in Figure 4A,B, it is evident that the highest ZP magnitude was just attained at the lowest concentration of fatty acid (A) for both types (D), yet at all surfactant concentrations (B) in case of OA-based vesicles or at the highest Span^®^ concentration for HDA vesicles.

#### 3.2.4. EE Response

The results obtained according to the adopted statistical design showed variable Mag EE reaching nearly complete entrapment (~97%) for drug-loaded formulae, as presented in Table 2. Based on the ANOVA results (Table 3), all independent factors, except the surfactant concentration (B), exhibited significant impacts on drug EE (*p* < 0.05).

##### Effect of Fatty Acid Concentration on EE

As illustrated in Figure 2E, the EE data revealed a parabolic profile by increasing fatty acid concentration (A) from 0.25 to 0.75%, as the highest EE occurred at mid-concentration (0.5%) in the case of OA-based ufasomes. This could be related to a bilayer domain saturation with the drug leading to a reduction in vesicle stability with possible drug leakage at the high OA concentration [27,28,57]. On the other hand, a directly proportional relation occurred between EE and HDA concentration, where the highest EE was scored at the highest concentration of fatty acid (0.75%), as shown in Figure 2F. These results revealed that the high concentrations of HDA enhanced vesicle stability and allowed for more drugs to be retained within the lipid bilayer, probably due to drug/lipid interaction via hydrogen bonding between the -OH groups of both Mag and fatty acid.

##### Effect of Drug Concentration on EE

Surprisingly the drug loading into ufasomes extremely mounted average EE to high values exceeding 80%. It is to be noticed that a non-significant EE was achieved between both drug concentrations used (C:10 and 20%) (*p* > 0.05), as depicted in Figure 2E,F.

##### Effect of Fatty Acid Type on EE

The EE was found to be dependent on the type of fatty acid (D). For instance, the alteration from OA to HDA was associated with a significant reduction in drug EE (*p* < 0.05), where the values for the respective average EE were 90 and 75%, as shown in Figure 2E,F. These results could be attributed to the structural difference of both fatty acids where OA is known for its ability to enhance the fluidity of lipid membranes [51], providing more flexibility to vesicles due to the presence of unsaturation and allowing for encapsulation of high drug loading. Furthermore, the small-sized HDA vesicles formed were believed to retain less drug compared to the larger OA vesicles. This is probably due to the greater curvature of the former vesicles with their looser packing between membrane lipids in the membranes, compared to OA vesicles [52].

##### Two-Way Interactions

The ANOVA results in Table 3 revealed only one significant 2-FI interaction between factors A and C (*p* < 0.05), as presented in Figure 4C,D. In the case of both types of fatty acid vesicles, the highest drug EE was reached at a Mag concentration equal to 10% or higher, irrespective of fatty acid concentration. This indicates that both fatty acids at the used concentrations had a high capacity to accommodate Mag concentrations up to 10% within their lipid bilayer without affecting the vesicle stability. Similar results were reported for dexamethasone when loaded in ufasomes at OA: drug ratio of 8:2 [34], also clotrimazole loaded ufasomes at OA: drug ratio of 6:4, methotrexate ufasomes at a ratio of 7:3; produced the highest EE and any further increase in drug reduced the entrapment which was attributed to reducing the vesicle stability by high drug concentrations [30,57].

##### 3.2.5. Model Validation

To validate the suggested models for the three evaluated responses, PS, ZP and EE, five new formulations were randomly chosen within the design space of the experiment and were prepared. The obtained experimental results were compared to the predicted ones, and then the prediction error (%bias) was calculated for each response model. The predicted data and their corresponding experimental ones, and the calculated prediction errors are collected in Table 5. As shown, the results of prediction error are all below 20%, confirming the validity and prediction capability of the three response models.

##### 3.2.6. Optimization Analysis

Numerical optimization was conducted to optimize the vesicular formulations based on some target goals; (1) minimize PS, (2) PDI and ZP within the data range, and (3) maximize drug EE. Three formulations were then chosen in accordance with the highest desirability function (D) obtained, which approached the unity. The compositions of the optimized formulations and their measured parameters are presented in Table 6. F-O1, F-O2 and F-O3 were the optimized formulations as their D values approached unity, and then they were selected for further characterization in the subsequent sections.

### 3.3. Characterization of the Optimized Fatty Acid Vesicles

#### 3.3.1. Differential Scanning Calorimetry (DSC)

**Figure 5:**
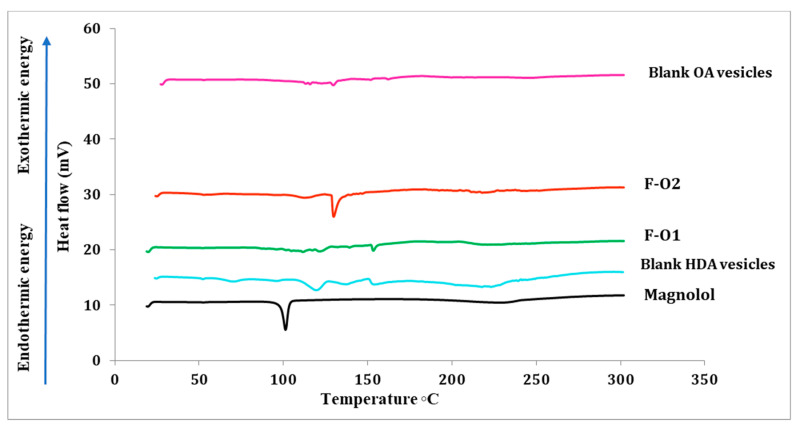
DSC thermograms of Mag, representative blank and loaded OA and HDA-based vesicles.

DSC is a thermo-analytical technique commonly applied to illustrate the recrystallization, melting behavior and thermodynamic properties of drug molecules. It depends on detecting any changes while exposing materials to a controlled heat flux [26]. As illustrated in Figure 5, the DSC thermogram of Mag shows a characteristic endothermic peak at 100 °C consistent with its reported melting point [17]. No specific thermal behavior was observed for plain OA ufasomes. Conversely, the plain HDA vesicles revealed a characteristic endothermic peak at 113 °C, most probably attributed to the HDA melting point, which is supposed to be around 88 °C [53]. The shifted melting peak of HDA could be explained by the presence of other components in the vesicles, such as span^®^80 and Tris buffer [54]. The DSC thermogram of the optimized drug-loaded vesicles (F-O2) revealed the appearance of an HDA characteristic endotherm. The disappearance of the Mag endothermic peak in the dried fatty acid vesicles (F-O1 and F-O2) confirms the drug amorphization and its entrapment and internal arrangement in the vesicles [24,26].

#### 3.3.2. TEM Imaging

**Figure 6:**
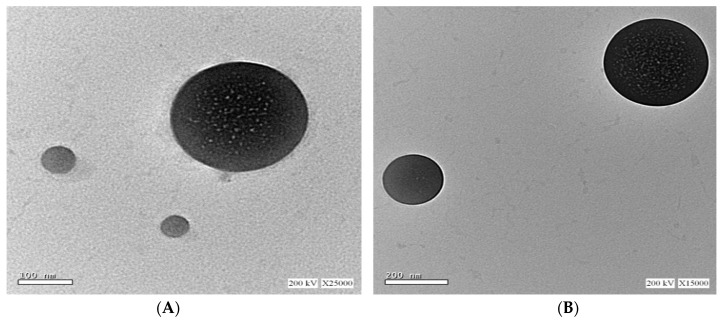

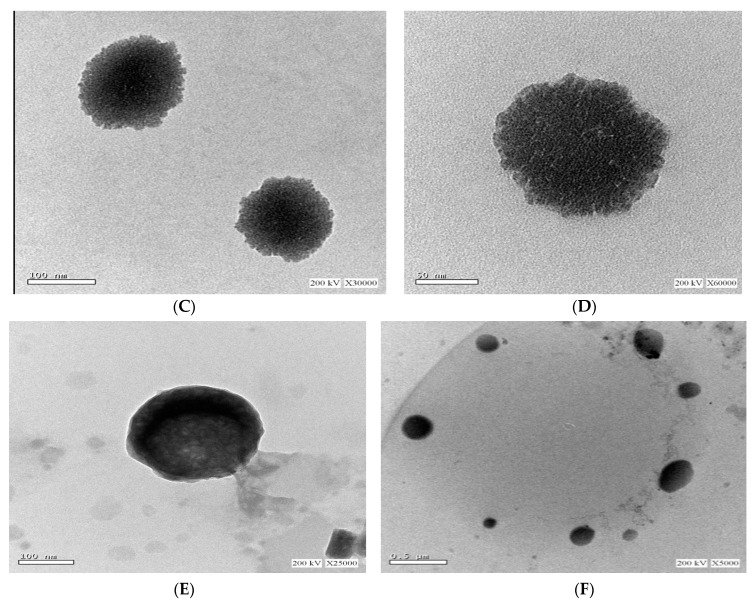
TEM imaging of (**A**,**B**) F-O1, (**C**,**D**) F-O2, and (**E**,**F**) F-O3, each at two different magnification powers.

As shown in Figure 6A–C, the optimized vesicle dispersions, F-O1, F-O2 and F-O3, show non-aggregated vesicles of nearly spherical shape. The observed vesicle sizes were found to be smaller than those measured by the DLS technique. This could be ascribed to the drying process to which the vesicles were subjected before imaging [24]. As shown in Figure 6A, the OA vesicles were more defined and had sharper edges when compared to HDA (Figure 6B,C). The rough surface of F-O2 shown in Figure 6B could be attributed to the high concentration of surfactant deposited on the surface of vesicles. Figure 6C shows a more defined surface of F-O3 formulation which could be attributed to the higher amount of HDA and lower amount of surfactant in F-O3 compared to F-O2.

### 3.4. In Vitro Drug Release Studies

**Figure 7:**
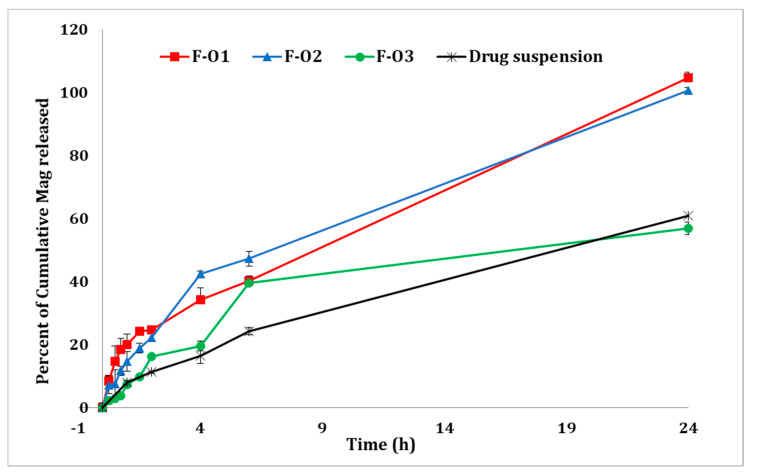
In vitro release profiles of Mag from different optimized fatty acid vesicles and free drug suspension in PBS (pH 7.4) containing 2%*w*/*v* Tween 80^®^ at 37 °C.

The in vitro Mag release study was conducted on each of the optimized fatty acid vesicles (F-O1, F-O2, and F-O3) and the aqueous drug suspension as well, using our own designed diffusion cells through a dialysis membrane for 24 h. The percentage of drug released was calculated at different time intervals for each formula and was then plotted to construct the corresponding release profile. The results are illustrated in Figure 7.

Referring to the different release data obtained, it can be seen that F-O1 and F-O2 formulations showed close release profiles with complete drug release achieved during the 24 h. Moreover, lower Mag release profiles were observed with both F-O3 and the drug suspension in comparison to the former formulae, where only 55–60% of the drug dose was released during the 24-h experiment. The higher Mag EE in F-O3 (92.6 ± 1.20 %), delineating that the drug was almost completely entrapped within the vesicles, explains the lower burst release noted (after 15 min) and the lower cumulative release in 24 h compared to the other two formulae.

### 3.5. Ex Vivo Skin Permeation Studies

**Figure 8:**
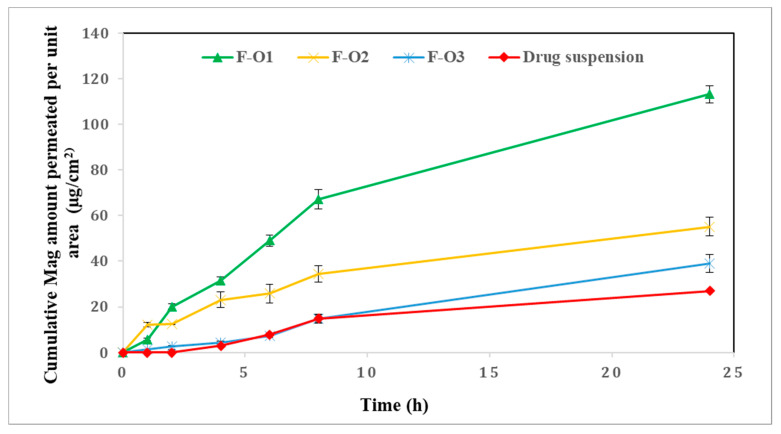
Ex vivo Mag permeation profiles through the skin from the optimized fatty acid vesicles and drug suspension.

The skin permeation study was performed on optimized formulations (F-O1, F-O2, F-O3) and free drug dispersion across excised rat skin for 24 h. The plots of the cumulative amount of drug permeated per unit area (μg/cm^2^) as a function of time are illustrated in Figure 8. A significant enhancement (*p* < 0.05) in drug permeation was recorded for OA vesicles (F-O1) in comparison to both HDA vesicles (F-O2 and F-O3) and drug suspension, where the respective amounts of Mag permeated after 24 h (Q_24_) were 113.24 ± 6.39, 55.19 ± 6.97, 39.03 ± 6.80 and 26.91 ± 4.50 µg/cm^2^ in case of F-O1, F-O2, F-O3, and free drug dispersion. This enhanced permeation for F-O1 could be attributed to the previously reported conspicuous penetration enhancer effect of OA [24,51,55,56]. The discrepancy in amounts of drug permeated between both HDA-based vesicles (F-O2 and F-O3) can probably be referred to as the difference in their PS. Both drug suspension and formula F-O3 showed low permeation profiles till 8 h, after which F-O3 scored a higher amount of Mag permeated (39.03 ± 6.80 µg/cm^2^) at 24 h compared to the former formula (26.91 ± 4.50 µg/cm^2^).

The different permeation parameters were also determined and collected in Table 7. The slope of the linear portion of the graph was used to compute the steady-state flux (Jss) [33]. The obtained results can be arranged in descending order as follows; F-O1 > F-O2 > F-O3 > drug suspension, confirming the constructed permeation profiles. The enhancement ratio (ER) was also calculated taking the flux of the drug suspension as a reference control and was found to be significantly higher in the case of OA vesicles (F-O1, ER= 2.24) compared to the other tested formulae, F-O2 and F-O3 scoring 1.72 and 1.39, respectively. These results could be related to the high ability of OA to enhance drug permeation through the skin by causing fluidization of membranes and disruption of the stratum corneum [58,59]. F-O3 resulted in a lower ER compared to F-O2, which could be attributed to the higher content of HDA in F-O3, which is responsible for the production of larger PS with consequent lower permeation compared to F-O2.

### 3.6. Skin Deposition Studies

**Figure 9:**
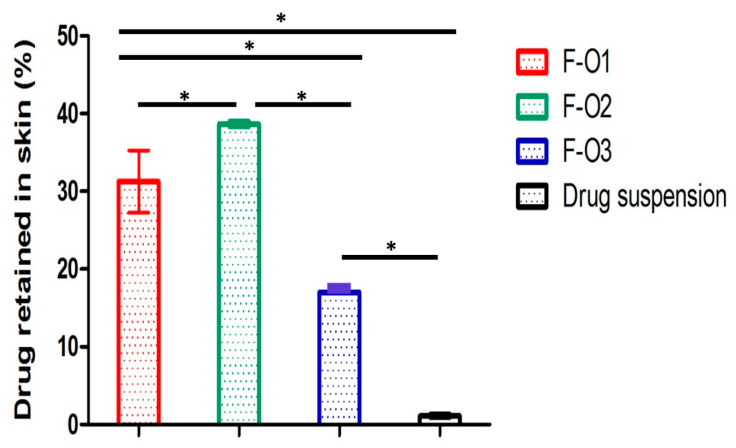
Percent Mag deposited in the skin from different optimized ufasomal formulations and drug suspension. * Significant difference (*p* < 0.05).

By analyzing the percentage of drug deposited in the skin after 24 h contact time, the results, illustrated in Figure 9**,** reveal that F-O2 showed the highest percentage of drug retained (38.66 ± 0.04%) compared to the other two vesicular formulae (F-O1; 31.25 ± 5.07%) and (F-O3; 17.02 ± 0.09%) at (*p* ≤ 0.05). F-O3 scored a significantly lower % of drug retained in the skin compared to F-O1, besides its lower flux, probably due to its larger size, which hindered or slowed its skin penetration. As expected, the drug suspension exhibited a significantly lower percentage of drug deposited in the skin (*p* < 0.05) (1.12 ± 0.27%) compared to the fatty acid vesicles.

These results elected HDA-based vesicles (F-O2) as a suitable topical nano-carrier system due to their high skin deposition and good permeation comparable to OA-based vesicles. Subsequent in vivo studies are needed to confirm this finding.

### 3.7. Physical Stability Study

**Figure 10:**
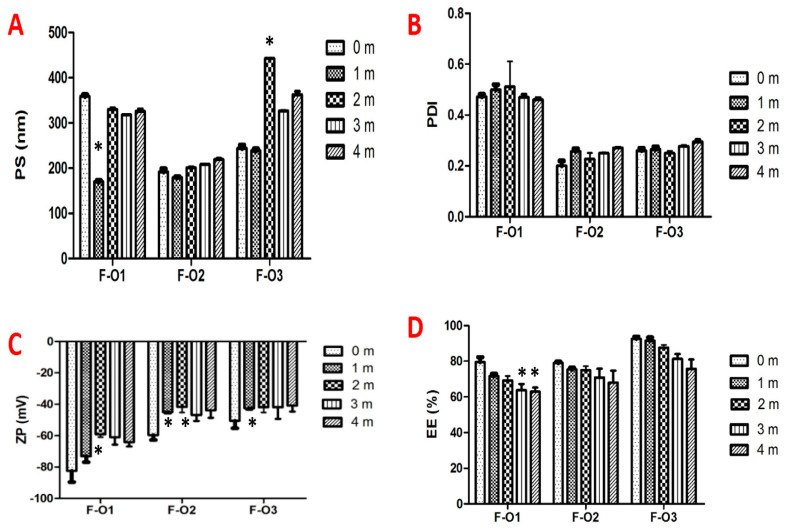
Physical stability results of the optimized ufasomal formulations (F-O1, F-O2 and F-O3), in terms of (**A**) PS, (**B**) PDI, (**C**) ZP, and (**D**) EE, after storage under refrigeration for 4 months. * Significant difference (*p* < 0.05) compared to initial data (0 m).

The three optimized vesicular formulations (F-O1, F-O2, F-O3) were stored under refrigeration for 4 months. The PS, PDI, ZP, and EE were determined for each formula at a one-month interval. The stability data of the three selected formulations are presented in Figure 10. All formulations were considered stable for 4 months, as the majority of measured parameters remained unchanged. However, the drug EE% significantly decreased after storage, mostly ascribed to the desorption of the drug physically adsorbed onto vesicle surfaces [28].

### 3.8. In Vivo Assessment

The optimized fatty acid vesicle dispersions, F-O1, F-O2 and F-O3, have been selected based on the following criteria: the smallest vesicle size, the highest drug entrapment efficiency, in addition to their promising results of ex vivo drug permeation and skin deposition, they were, thus, believed in providing better treatment. Therefore, these formulations were included in the in vivo studies for comparison of their effectiveness in the treatment of skin cancer and were given to Groups III, IV and V, respectively, in comparison to the negative and positive controls, Groups I and II. OA was the building carrier in F-O1, while HDA was in F-O2 and F-O3. F-O2 was also selected to test the chemo-preventive effect of Mag in the prophylactic group (VI).

#### 3.8.1. Mice Body Weight

The mice selected for the study weighed 20–25 g, and their weights were determined initially and were then monitored during the entire duration of the study.

Four weeks following the beginning of induction, papillomas started to appear in Groups II to V, but the mice’s body weight was not significantly affected. Conversely, Group VI, which was pretreated with F-O2, did not develop any obvious papillomas.

No significant differences were found during the first weeks of treatment in all groups. As shown in Table 8, significant reductions in the average body weight of mice in Groups II, III, and IV (*p* < 0.05) were noticed compared to Group I (negative control) at the end of the study. The prophylactic Group VI is considered the lowest group affected in terms of body weight as it showed an average weight of 22.06 ± 0.87 g after 30 days of treatment compared to its initial value of 24.09 ± 1.01g. Additionally, Group V of mice applying the HDA-vesicular dispersion F-O3 revealed non-significant changes in average body weight (*p* > 0.05) where the recorded data was 23.13 ± 1.88 and 20.04 ± 1.63 g at time 0 and 30 days of treatment, respectively. These results reflected the effectiveness of treatments administered to these groups.

#### 3.8.2. Tumor Size and Number of Papilloma

**Figure 11:**
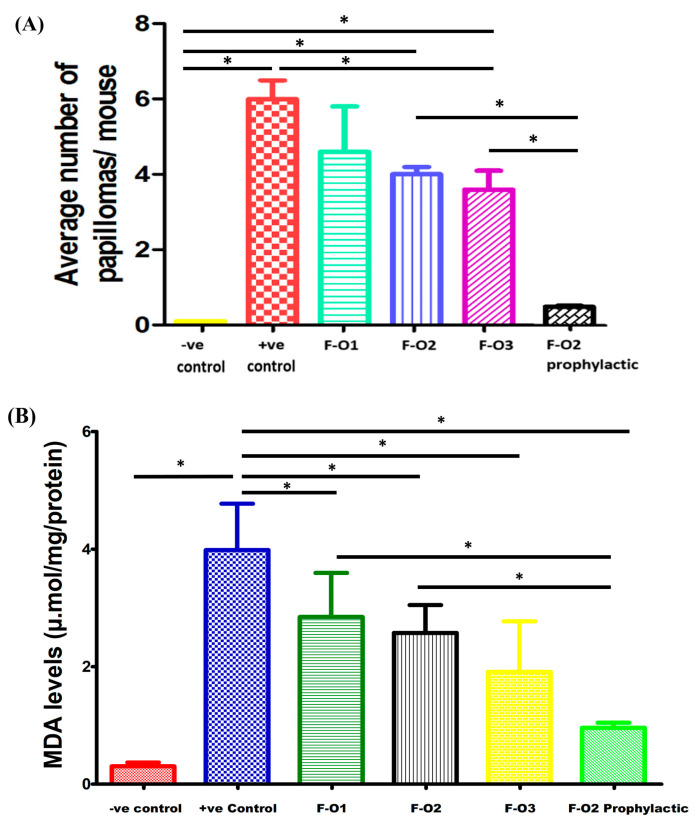

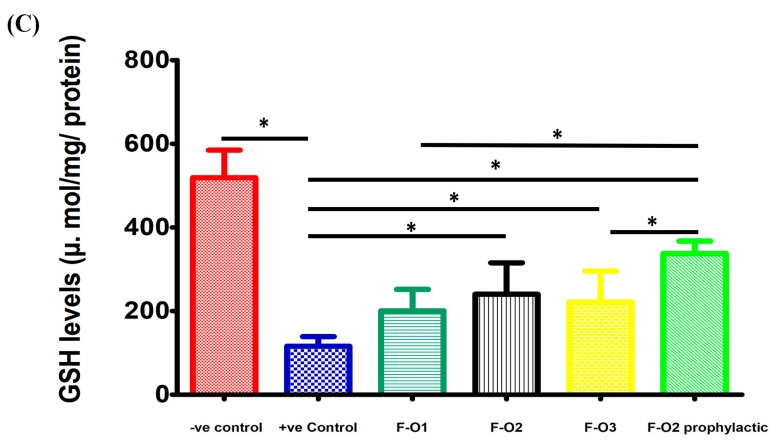
(**A**) An average number of papilloma/mice for each treatment group was recorded at the end of the study. (**B**) MDA levels in skin samples of different mice groups. (**C**) GSH levels in skin samples of different mice groups. * Significant difference (*p* < 0.05).

Tumor incidence and an average number of papillomas were recorded weekly until the end of the experiment; the outgrowth of > 1mm in diameter was considered positive tumor formation [13,60]. The tumor incidences in different mice groups are shown in Figure 11A.

**Figure 12:**
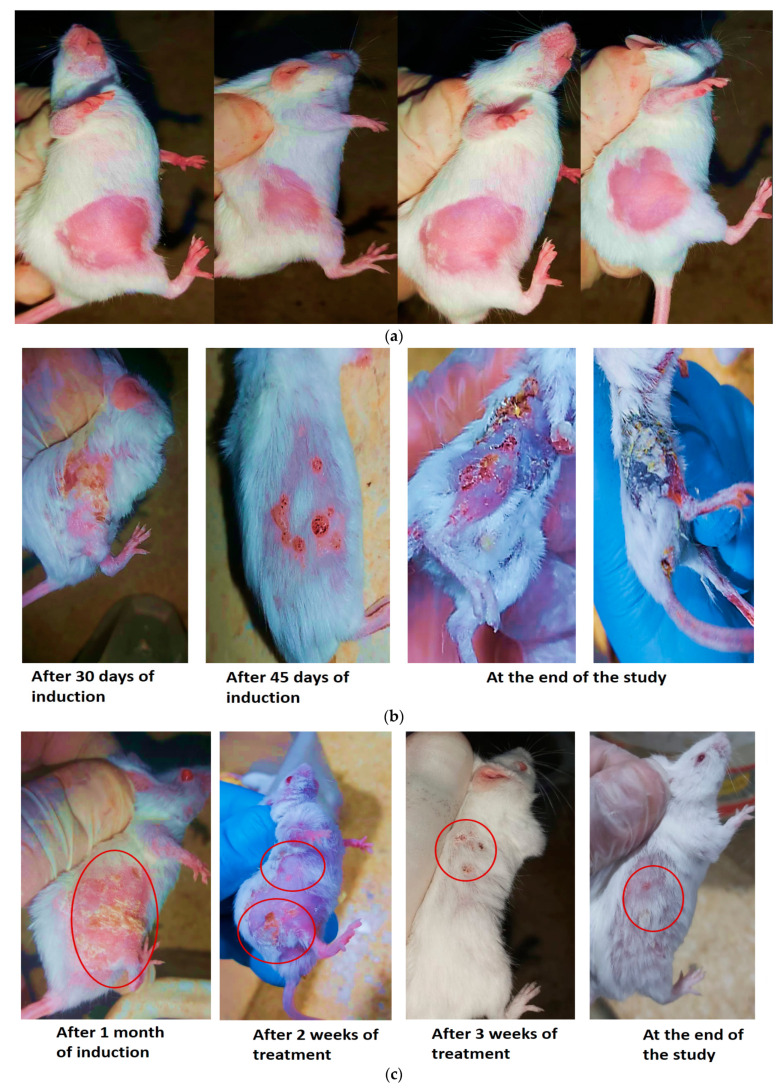

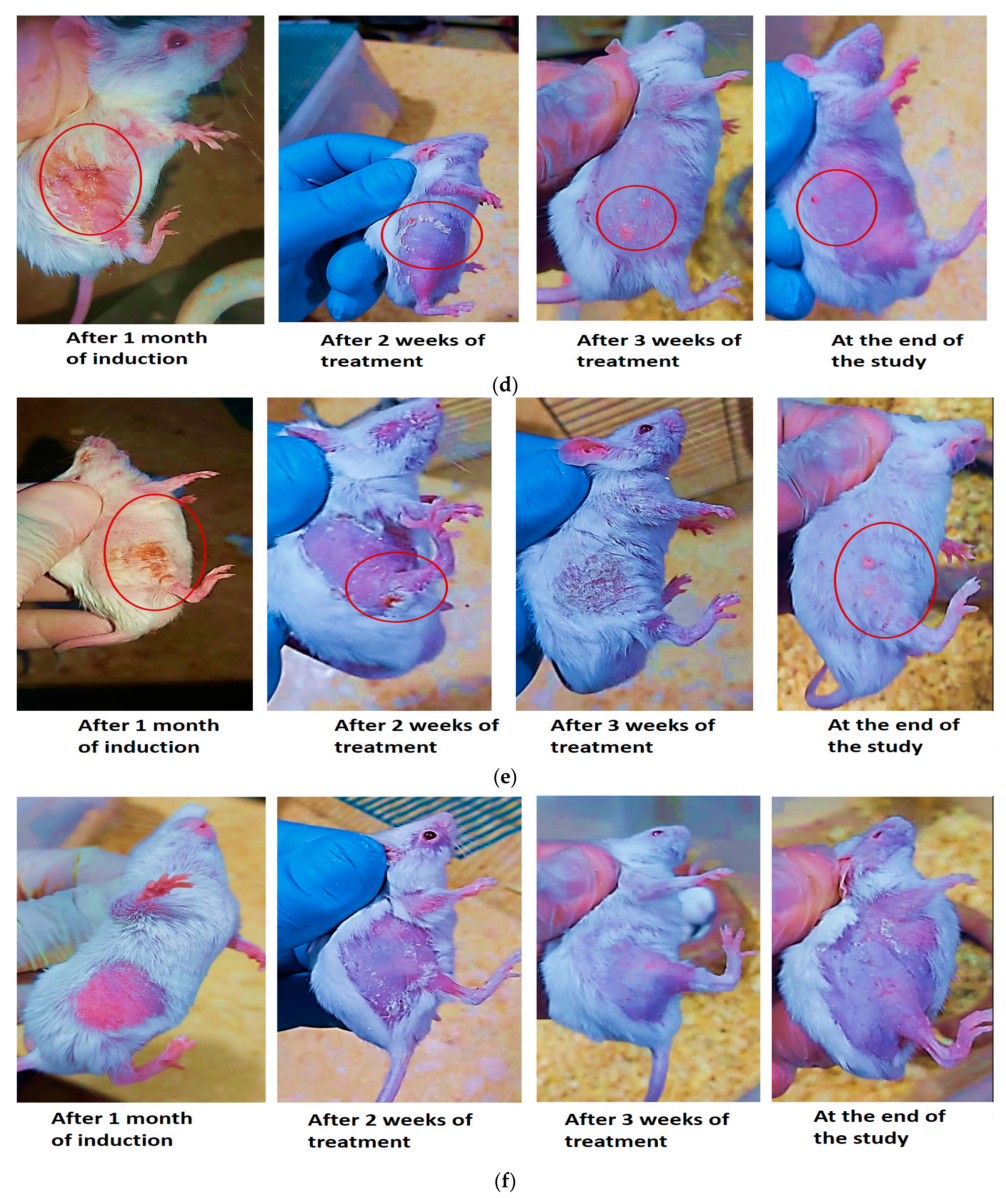
Representative images of DMBA-induced skin cancer recorded for mice of different groups at different time intervals: (**a**) Group I (negative control), (**b**) Group II (positive control), (**c**) Group III (F-O1), (**d**) Group IV (F-O2), (**e**) Group V (F-O3), and (**f**) Group VI (prophylactic treatment with F-O2).

After 4 weeks of induction, papillomas with an average diameter of 1.5 ± 0.06 mm started to appear in Groups II to V. As shown in Figure 12b, mice of Group II (positive control) developed multiple spherical papillomas with severe inflammation in the skin and hard crust surrounding papillomas. During the study, the papillomas became more severe compared to all groups receiving treatment and led to significant deterioration in animal health with subsequent death.

Group II (positive control) scored the highest number of papillomas (6.7 ± 0.5 mm) per mouse, as shown in Figure 11A. Group VI, pre-treated with F-O2, did not develop any obvious signs of tumor growth until week 6 from the beginning of induction. Only mild inflammation could be observed in certain mice of this group. Lower tumor multiplicity was significantly demonstrated (*p* < 0.05) in Group VI compared to the positive control group and other treatment groups as it scored the lowest average number of papilloma per mouse of <0.5 ± 0.08 in only 5 mice out of 15 at the end of the study as presented in Figure 11A and Figure 12f. These results confirmed the effectiveness of Mag as a chemo-preventive agent against skin cancer. Similar results were reported by [14], who demonstrated that Mag induces apoptosis and reduces cell proliferation through the modification of various signaling pathways.

As obvious in Figure 11A, both HDA-based vesicles (F-O2 and F-O3) revealed a significantly lower number of papillomas (*p* < 0.05) when compared to the positive control, while OA-based vesicles (F-O1) showed a non-significant difference (*p* > 0.05).

After 2 weeks of treatment, Figure 12d,e reveals that Groups IV and V treated with F-O2 and F-O3, respectively, showed significant improvement in skin condition manifested as reduced inflammation and a decrease in the number and size of papillomas. On the other hand, Group III, treated with F-O1, illustrated in Figure 12c, did not show marked improvement, although no further deterioration occurred. Groups IV and V, Figure 12d,e, show further enhancement in overall skin condition; regrowth of hair with no skin inflammation could be observed. Group III, Figure 12c, started to show improvement compared to previous weeks of treatment, manifested as a marked reduction in the size of papillomas with obvious hair growth. The obtained results are ascribed to the enhanced permeability of Mag from HDA vesicles and also confirm the synergistic effect of HDA as an anti-cancer [61].

#### 3.8.3. MDA and GSH Levels in Skin Tumor Samples

To provide a proof of concept of the anti-cancer activity of the selected formulations, MDA and GSH levels were assessed in skin samples excised at the end of the study (after 30 days of treatment). The values obtained for each marker are illustrated in Figure 11B,C.

Low levels of MDA in skin samples indicate reduced oxidative stress [39], hence a more effective treatment for skin cancer. The groups that received post-induction treatments (without prophylaxis) showed a descending order of effectiveness as follows; F-O3 > F-O2 > F-O1, where the respective MDA levels were 1.91 ± 0.86, 2.58 ± 0.47 and 2.8 ± 0.75 µmol/mg of protein as shown in Figure 11B. Only Group III receiving F-O1 showed a non-significant reduction in MDA levels (*p* > 0.05) when compared to Group II (positive control), which reflected poor efficacy in the treatment of skin cancer. In contrast, Groups IV and V, treated with F-O2 and F-O3, respectively, showed a significant reduction in MDA levels (*p* < 0.05) compared to Group II (positive control), which proved their effectiveness in the management of DMBA-induced skin cancer and confirmed the additional influence of HDA in defeating skin cancer. However, Group VI (prophylactic) showed the lowest MDA levels of 0.97 ± 0.08 µmol/mg of protein Figure 11B, which were considered non-significantly different than that of the negative control (*p* > 0.05). These results confirmed the effectiveness of Mag-HDA vesicles as a prophylactic treatment and endorsed the synergistic anti-cancer effect of HDA as reported [49].

GSH is a substantial antioxidant, and its expression at high levels indicates effective treatment for cancer [40]. GSH expression data were also consistent with MDA results. Group VI receiving prophylactic treatment showed a significant increase in GSH levels (316.00 ± 58.90 µmol/mg of protein) (*p* < 0.05) compared to the other groups, as seen in Figure 11C. It could be noted from Figure 11C that GSH levels can be arranged as follows; F-O2 > F-O3 > F-O1. Groups IV and V, treated with F-O2 and F-O3, respectively, showed a significant increase in GSH levels (*p* < 0.05) compared to Group II (positive control), which proves their effectiveness in skin cancer treatment. These superior results of HDA-based formulations could be attributed to the synergistic antioxidant and antitumor effects of HDA [49].

#### 3.8.4. Immunohistochemistry (Ki-67 Expression) Analysis

**Figure 13:**
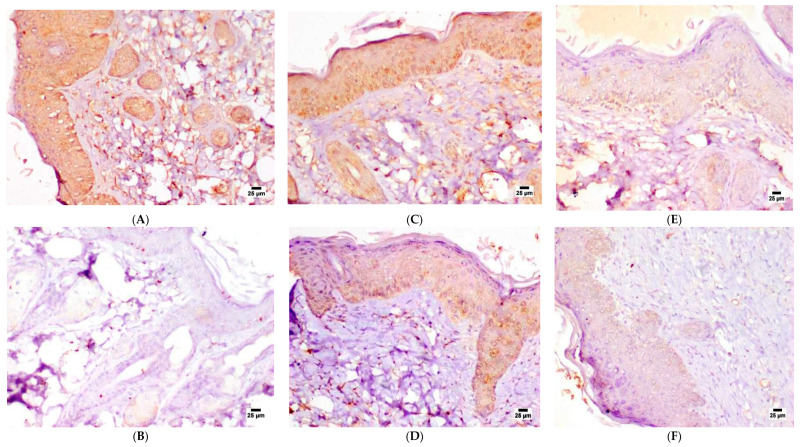
Photomicrographs of mice skin sections of (**A**) Group I (negative control), (**B**) Group II (positive control), (**C**) Group III (F-O1), (**D**) Group IV (F-O2), (**E**) Group V (F-O3) and (**F**) Group VI (prophylactic F-O2) (immune staining).

Immune expression of Ki-67 in the epidermis of Group I (negative control) was shown in Figure 13A, demonstrating negative expression in the basal cell layer. Intense positive staining was noticed in all examined sections of Group II (positive control), as seen in Figure 13B, where immune staining was distributed in the epidermis and the proliferating neoplastic cells in the dermis. The images of Group III (F-O1) presented in Figure 13C showed only a mild reduction in Ki-67 expression compared to the positive control group (Group II). Positive immune staining of Ki-67 in sections treated with F-O2 (Group IV) was moderate to mild in the epidermal layer, as obvious in Figure 13D. A marked reduction in Ki-67 expression was noticed in Group V (F-O3), shown in Figure 13E. The least expression of Ki-67 was detected in Group VI receiving prophylactic treatment of (F-O2), as obvious in Figure 13F. The obtained results delineated the superiority of HDA-based formulations, F-O3 and prophylaxis with F-O2 in attenuating DMBA-induced skin cancer. This could be attributed to the synergistic antioxidant and antitumor effects of HDA [49].

#### 3.8.5. Histopathological Examinations

**Figure 14:**
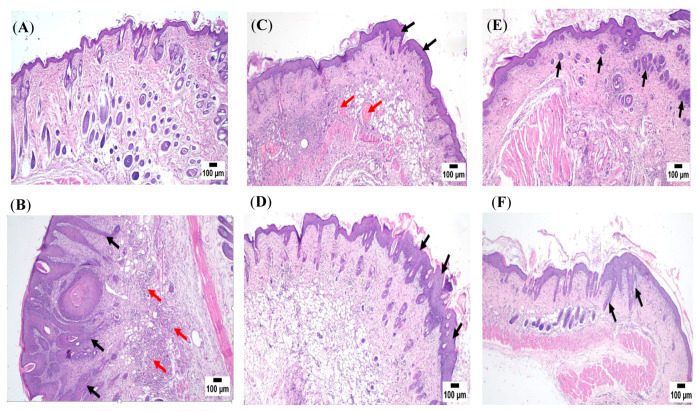
Skin photomicrographs of (**A**) Group I (negative control), (**B**) Group II (positive control) showing downward projections of neoplastic cells into the underlying dermis (black arrows), Intense mononuclear inflammatory cells infiltration at the dermal layer (red arrows), (**C**) Group III (F-O1) showing mild thickening in the epidermis (black arrows) with diffuse inflammatory cells infiltration and numerous severely dilated blood vessels at the dermis (red arrows), (**D**) Group IV (F-O2) showing thickened epidermis with downward growths (black arrows) with diffuse inflammatory cells infiltration in the dermis, (**E**) Group V (F-O3) showing acanthosis with separation of neoplastic cells forming bird nest appearance (black arrows) and (**F**) Group VI (prophylactic F-O2) showing small epidermal projections with diffuse inflammatory cells infiltration (black arrows) (H&E staining).

Microscopic examination of skin sections of Group I (negative control) illustrated in Figure 14A revealed the normal structure of skin consisting of an upper epidermis layer that appeared composed of multiple cell layers including basal cell layer, brickle cell layer, granular cell layer and keratin layer. The epidermis rested on the dermis layer formed of connective tissue that contained hair follicles, sebaceous and sweat glands.

In contrast, the histopathological examination of Group II (positive control) skin specimens presented in Figure 14B showed serious alterations; the epidermis was greatly thickened, forming finger-like projections headed upward with downward extension into the underlying dermis. The proliferating neoplastic cells penetrated through the basement membrane and invaded the dermis with the formation of separated groups giving the characteristic bird nest or epithelial pearls of squamous cell carcinoma, in which the neoplastic cells form groups with central keratin whorl. Those cells exhibited the criteria of malignancy, including hyperchromasia, anisokaryosis and frequent atypical mitosis. The basal cell layer of the epidermis showed marked dysplasia with frequent mitotic figures. The dermal layer suffered extensive diffuse inflammatory cell infiltration. Some severely affected cases showed deep invasion of the dermis by poorly differentiated neoplastic cells with multinucleated giant tumor cells formation.

Skin images of Group III treated with (F-O1) exhibited mild improvement, as noticed in Figure 14C; the histological picture included focal thickening of the epidermis with marked dysplasia and frequent mitosis at the basal cell layer. Mild dermal edema was also observed. Some severely affected sections showed multiple finger-like projections of the epidermis, proliferation in basal cells and invasion of the dermis by small groups of neoplastic cells, as well as inflammatory reaction at the dermis. Formation of the characteristic epithelial bird structure of invading cells was observed as well. Concerning Group IV treated with F-O2, the skin images in Figure 14D noticed only mild improvement, represented by focal acanthosis in the epidermis with diffuse inflammatory reaction in the dermis. The basal cell layer showed dysplasia and frequent mitosis. The inflammatory reaction at the dermis existed too. A better protective action was noticed in skin sections of Group V (F-O3), as an apparently normal epidermis was observed in many sections with mild dysplasia at the basal cell layer and mild dermatitis, as shown in Figure 14E. A few sections showed small downward growths of neoplastic cells extending into the dermis. One severely affected case showed separated groups of neoplastic cells with central keratin whorl forming the bird’s nest appearance.

The best antitumor action was observed in skin sections of Group VI (prophylactic group), demonstrated in Figure 14F; the detected lesions were represented mainly by acanthosis with downward growths of the epidermis. Mild dysplastic changes were noticed in the basal cell layer. Dermatitis was mild and focal in distribution. The invading neoplastic cells were hardly detected in the upper dermal layer. 

To recapitulate, it was proved that Group VI, which received F-O2 as a prophylactic treatment, showed the best anti-tumor action, which confirms the effectiveness of Mag-loaded HDA vesicular suspension as a chemo-preventive agent for DMBA-induced skin cancer. The anti-tumor activity of HDA was reported in literature [62,63,64], which contributed to the enhancement of the Mag effect in the previously mentioned formulations. Moreover, Group V treated with F-O3 demonstrated the best treatment options confirming the superiority of Mag loaded into HDA-based vesicles containing Mag in defeating skin cancer due to the reported anti-cancer activity of the fatty acid (HDA) in both formulae.

## 4. Conclusions

Innovative HDA-based vesicles were successfully prepared by the thin film hydration technique compared to conventional OA ufasomes. The fatty acid vesicles were optimized using statistical Box–Behnken design where PS and PDI ranged between 156.40 ± 1.56 and 836.60 ± 397.90 nm, 0.168 ± 0.014 and 0.790 ± 0.231. High negative magnitudes of ZP values were attained, varying from −44.1 ± 7 to −90.1 ± 3 mV. All Mag-loaded formulations showed high EE of more than 63% to almost complete drug loading (~97%). Ex vivo permeation studies revealed enhanced permeation of Mag from the optimized vesicle formulations; F-O1 (OA-based) > F-O2 (low concentration HDA-based) > F-O3 (high concentration HDA-based) compared to a drug suspension. Skin deposition experiments demonstrated that F-O2 provided the highest drug retention, which confirms its choice as a successful nanocarrier for topical delivery of Mag. In vivo, studies revealed the superiority of HDA-based formulation in attenuating DMBA-induced skin cancer as a treatment and chemo-preventive carrier. This was confirmed by the least immune Ki67 expression, least MDA levels, highest GSH levels, lower tumor multiplicity and best histopathological studies. The results of the study confirmed the superiority of Mag-loaded HDA-based vesicles over conventional OA ufasomes for the topical treatment of skin cancer.

## Figures and Tables

**Table 1:** Levels and independent variables of the Box–Behnken design constructed for the preparation of Mag-vesicles.

Independent Variable	Low Level (−1)	Medium Level (0)	High Level (+1)
A: Fatty Acid Concentration (%*w*/*v*)	0.25	0.5	0.75
B: Span^®^ 80 Concentration^(a)^ (%*w*/*w*)	5	12.5	20
C: Mag Concentration ^(a)^ (%*w*/*w*)	0	10	20
D: Fatty acid type ^(b)^	OA	-	HDA

^(a)^ Calculated based on fatty acid weight, ^(b)^ this categorical variable was tested at two levels only. Mag: Magnolol, OA: Oleic acid, HDA: 10-hydroxy decanoic acid.

**Table 2:** Independent variables and responses used for the preparation of fatty acid vesicles according to Box–Behnken Design.

Formula Code	Independent Variables				Responses * ± SD			
	A: Fatty Acid Concentration	B: Surfactant Concentration	C: Drug Concentration	D: Fatty Acid Type	Y_1_: PS (nm)	Y_2_: PDI	Y_3_: ZP (mV)	Y_4_: EE
F1	0.25	12.5	0	OA	662.75 ± 40.09	0.638 ± 0.110	−83.4 ± 1	0
F2	0.5	12.5	10	OA	557.40 ± 20.51	0.676 ± 0.168	−83.6 ± 5	95.08 ± 2.82
F3	0.25	12.5	20	HDA	198.17 ± 11.34	0.200 ± 0.008	−61.2 ± 2	63.86 ± 6.78
F4	0.75	12.5	0	HDA	413.20 ± 55.50	0.560 ± 0.122	−48.7 ± 1	0
F5	0.5	5	0	OA	547.85 ± 155.35	0.509 ± 0.038	−87.1 ± 6	0
F6	0.5	12.5	10	HDA	297.37 ± 14.72	0.360 ± 0.022	−57.6 ± 4	73.90 ± 4.87
F7	0.25	20	10	OA	362.30 ± 21.92	0.530 ± 0.098	−83.0 ± 2	68.44 ± 12.29
F8	0.5	5	20	OA	324.20 ± 36.20	0.568 ± 0.122	−82.7 ± 3	80.03 ± 3.01
F9	0.75	12.5	20	OA	532.07 ± 102.89	0.767 ± 0.224	−63.9 ± 2	84.15 ± 1.41
F10	0.5	12.5	10	HDA	304.90 ± 16.87	0.366 ± 0.211	−61.7 ± 5	70.82 ± 4.51
F11	0.5	5	20	HDA	752.80 ± 5.37	0.790 ± 0.231	−44.8 ± 3	86.55 ± 0.96
F12	0.25	20	10	HDA	185.80 ± 17.96	0.229 ± 0.120	−65.0 ± 4	65.37 ± 2.30
F13	0.25	12.5	20	OA	291.55 ± 49.55	0.539 ± 0.147	−90.1 ± 3	82.68 ± 6.02
F14	0.25	5	10	OA	344.37 ± 43.88	0.549 ± 0.167	−89.6 ± 6	73.87 ± 19.97
F15	0.5	20	20	HDA	235.80 ± 11.74	0.305 ± 0.013	−59.3 ± 2	74.10 ± 1.41
F16	0.5	12.5	10	OA	542.90 ± 19.40	0.766 ± 0.176	−73.7 ± 1	93.09 ± 2.83
F17	0.75	5	10	OA	495.60 ± 25.17	0.529 ± 0.033	−78.4 ± 1	82.06 ± 8.79
F18	0.5	20	0	HDA	199.50 ± 1.77	0.229 ± 0.011	−56.4 ± 2	0
F19	0.75	20	10	HDA	481.05 ± 47.87	0.647 ± 0.203	−48.2 ± 1	81.46 ± 10.49
F20	0.5	12.5	10	HDA	306.80 ± 8.98	0.334 ± 0.183	−59.6 ± 1	71.37 ± 2.77
F21	0.5	12.5	10	OA	557.40 ± 45.31	0.735 ± 0.157	−78.65 ± 6	97.08 ± 1.92
F22	0.75	5	10	HDA	248.30 ± 15.98	0.407 ± 0.103	−51.1 ± 4	83.03 ± 4.86
F23	0.5	20	20	OA	437.60 ± 11.40	0.697 ± 0.143	−76.2 ±3	89.67 ± 0.13
F24	0.5	20	0	OA	477.80 ± 10.04	0.619 ± 0.047	−77.9 ± 2	0
F25	0.5	5	0	HDA	167.90 ± 13.01	0.230 ± 0.005	−65.4 ± 5	0
F26	0.75	20	10	OA	432.53 ± 125.53	0.650 ± 0.164	−63.0 ± 6	76.69 ± 1.71
F27	0.75	12.5	0	OA	836.60 ± 397.90	0.505 ± 0.021	−67.0 ± 4	0
F28	0.75	12.5	20	HDA	243.20 ± 37.62	0.337 ± 0.022	−44.1 ± 7	89.76 ± 7.21
F29	0.25	12.5	0	HDA	156.40 ± 1.56	0.168 ± 0.014	−69.7 ± 4	0
F30	0.25	5	10	HDA	185.10 ± 7.88	0.213 ± 0.056	−45.7 ± 2	76.77 ± 7.68

* Mean of 3 determinations. OA: Oleic acid, HDA: 10-hydroxy decanoic acid, PS: Particle size, PDI: Polydispersity index, ZP: Zeta potential, EE: Entrapment efficiency, SD: standard deviation.

**Table 3:** ANOVA test results of all responses studied for the preparation of fatty acid vesicles according to Box–Behnken design.

Terms	Responses					
	PS		ZP		EE	
	*F*-Value	*p*-Value	*F*-Value	*p*-Value	*F*-Value	*p*-Value
Model	165.82 *	<0.0001	23.65 *	<0.0001	143.77 *	<0.0001
A	387.78 *	<0.0001	41.61 *	<0.0001	18.94 *	0.0014
B	29.54 *	0.0003	0.68 ^NS^	0.4188	3.06 ^NS^	0.1110
C	162.06 *	<0.0001	3.03 ^NS^	0.0977	1832.62 *	<0.0001
D	39.57 *	<0.0001	168.48 *	<0.0001	46.57 *	<0.0001
AB	6.83 *	0.0259	5.26 *	0.0334	0.85 ^NS^	0.3792
AC	212.04 *	<0.0001	0.19 ^NS^	0.6674	6.48 *	0.0291
AD	217.62 *	<0.0001	1.62 ^NS^	0.2190	3.98 ^NS^	0.0739
BC	21.76 *	0.0009	3.76 ^NS^	0.0675	0.07 ^NS^	0.7991
BD	19.02 *	0.0014	9.72 *	0.0057	2.55 ^NS^	0.1416
CD	319.84 *	<0.0001	2.19 ^NS^	0.1551	2.14 ^NS^	0.1738
A^2^	19.54 *	0.0013			12.20 *	0.0058
B^2^	108.1 *	<0.0001			6.80 *	0.0262
C^2^	69.57 *	<0.0001			799.74 *	<0.0001
ABD	11.97 *	0.0061			0.83 ^NS^	0.3848
ACD	338.1 *	<0.0001			5.16 *	0.0464
BCD	48.24 *	<0.0001			4.23 ^NS^	0.0670
A^2^D	6.19 *	0.0321			14.81 *	0.0032
B^2^D	59.89 *	<0.0001			17.81 *	0.0018
C^2^D	55.21 *	<0.0001			6.46 *	0.0293
Lack of fit	3.03 ^NS^	0.1514	1.75 ^NS^	0.3128	1.07 ^NS^	0.4961

* Significant at 5% probability (*p* < 0.05) ^NS^ non-significant. A: Fatty acid concentration, B: Surfactant concentration, C: Drug concentration, D: Fatty acid type, PS: Particle size, ZP: Zeta potential, EE: Entrapment efficiency.

**Table 4:** Statistical analysis results of the studied responses according to Box–Behnken design.

Response	PS	ZP	EE
Suggested Model	Reduced Cubic	2FI	Reduced Cubic
Equation	(PS)^−3^ = +2.211 × 10^−8^ − 3.128 × 10^−8^A − 8.632 × 10^−9^B − 2.022 × 10^−8^C + 1.632 × 10^−8^D − 5.870 × 10^−9^AB + 3.271 × 10^−8^AC − 2.343 × 10^−8^AD + 1.048 × 10^−8^BC − 6.926 × 10^−9^BD − 2.841 × 10^−8^CD + 1.033 × 10^−8^A^2^ + 2.431 × 10^−8^B^2^ + 1.950 × 10^−8^C^2^ − 7.771 × 10^−9^ABD + 4.130 × 10^−8^ACD + 1.560 × 10^−8^BCD + 5.816 × 10^−9^A^2^D + 1.809 × 10^−8^B^2^D + 1.737 × 10^−8^C^2^D	ZP = −67.23 + 7.71A + 0.9875B + 2.08C + 11.33D + 3.88AB + 0.7375AC − 1.52AD − 3.28BC − 3.72BD + 1.77CD	EE = +84.49 + 4.13A − 1.66B + 40.68C − 10.59D + 1.24AB + 3.42AC + 1.90AD − 0.3512BC − 1.52BD − 1.39CD − 4.88A^2^ − 3.65B^2^ − 39.55C^2^ + 1.22ABD + 3.05ACD − 2.76BCD + 5.38A^2^D + 5.90B^2^D + 3.55C^2^D
R^2^	0.9968	0.9256	0.9964
Adjusted R^2^	0.9908	0.8865	0.9894
Predicted R^2^	0.9572	0.7965	0.9609
Adequate Precision	51.3279	15.0279	31.3583

A: Fatty acid concentration, B: Surfactant concentration, C: Drug concentration, D: Fatty acid type, PS: Particle size, ZP: Zeta potential, EE: Entrapment efficiency, 2FI: two-factor interaction, R^2^: coefficient of determination.

**Table 5:** Experimental data predicted data and prediction error (%) of PS, ZP and EE responses of the five randomly selected ufasomal formulations.

Formula Code	Formulation Composition	Experimental Results *			Predicted Results			Prediction Error (%)		
		PS (nm) ± SD	ZP (mV) ± SD	EE (%) ± SD	PS (nm)	ZP (mV)	EE (%)	PS	ZP	EE
F-V1	0.25% *w*/*v* OA, 6.96% *w*/*w* Span^®^80, and 14.56% *w*/*w* Mag.	358.90 ± 3.20	−82.50 ± 7.13	79.56 ± 2.76	310.96	−87.48	86.71	15.42	5.69	8.25
F-V2	0.47% *w*/*v* OA, 20% *w*/*w* Span^®^80, and 8.78% *w*/*w* Mag.	404.40 ± 10.54	−70.30 ± 6.20	88.97 ± 1.92	496.22	−74.56	79.11	18.50	5.71	12.46
F-V3	0.25% *w*/*v* HDA, 20% *w*/*w* Span^®^80, and 12.94% *w*/*w* Mag	191.90 ± 6.28	−59.60 ± 3.07	78.94 ± 0.86	200.37	−68.75	70.62	4.23	13.31	11.78
F-V4	0.75 % *w*/*v* HDA, 16.95 *w*/*w* Span^®^80, and 16.12% *w*/*w* Mag.	287.60 ± 11.48	−53.10 ± 4.78	93.18 ± 3.42	304.57	−47.42	94.19	5.57	11.98	1.07
F-V5	0.425 % *w*/*v* HDA, 15.69% *w*/*w* Span^®^80, and 15.35% *w*/*w* Mag	243.90 ± 6.61	−50.60 ± 4.60	92.62 ± 1.20	300.00	−58.23	79.83	18.70	13.10	16.02

* Mean of 3 determinations. OA: Oleic acid, HDA: 10-hydroxy decanoic acid, PS: Particle size, ZP: Zeta potential, EE: Entrapment efficiency, SD: Standard deviation. SD: standard deviation.

**Table 6:** Compositions and characteristics of the optimized fatty acid vesicular formulations.

Formula Code	Independent Factors				Measured Responses * ± SD			
	A: Fatty Acid Concentration (%*w*/*v*)	B: Surfactant Concentration (%*w*/*w*) ^a^	C: Drug Concentration (%*w*/*w*) ^a^	D: Fatty Acid Type	PS (nm)	PDI	ZP (mV)	EE (%)
F-O1	0.25	6.96	14.56	OA	358.90 ± 3.20	0.472 ± 0.011	−82.50 ± 7.13	79.56 ± 2.76
F-O2	0.25	20	12.95	HDA	191.90 ± 6.28	0.200 ± 0.021	−59.60 ± 3.07	78.94 ± 0.86
F-O3	0.425	15.69	15.35	HDA	243.90 ± 6.61	0.260 ± 0.013	−50.60 ± 4.60	92.62 ± 1.20

* Results are expressed as the mean of three determinations. **^a^** Calculated with respect to fatty acid weight. OA: Oleic acid, HDA: 10-hydroxy decanoic acid, PS: Particle size, PDI: Polydispersity index, ZP: Zeta potential, EE: Entrapment efficiency, SD: Standard deviation.

**Table 7:** Permeation parameters of the optimized fatty acid vesicles.

Formula Code	Q_24_^a^ ± SD (µg/cm^2^)	Jss (µg/cm^2^/h)	ER ^b^
F-O1	113.24 ± 6.39	4.61	2.24
F-O2	55.19 ± 6.97	2.05	1.72
F-O3	39.03 ± 6.80	1.66	1.39
Drug suspension	26.91 ± 4.50	1.19	---

^a^ Mean of 3 determinations SD: standard deviation. ^b^ calculated based on the drug suspension as a reference. Q_24_: Cumulative drug amount permeated per unit area after 24 h, Jss: Steady state transdermal flux; ER: Enhancement ratio.

**Table 8:** The average body weights of mice were measured from different groups initially and at the end during the in vivo anti-cancer studies.

Group Number	Initial Average Mice Body Weight (g) ± SD	Average Weight (g) at the End of the Study ± SD
I	23.00 ± 1.83	23.00 ± 1.83
II	23.03 ± 1.61	16.05 ± 2.01
III	24.07 ± 1.50	18.65 ± 0.79
IV	23.42 ± 1.67	19.55 ± 1.85
V	23.13 ± 1.88	20.04 ± 1.63
VI	24.09 ± 1.01	22.06 ± 0.87

Group I: negative control, Group II: Positive control, Group III: F-O1, Group IV: F-O2, Group V: F-O3, Group VI: prophylactic treatment with F-O2.

## Data Availability

The datasets generated during the current study are available from the corresponding authors upon request.
